# NWB Query Engines: Tools to Search Data Stored in Neurodata Without Borders Format

**DOI:** 10.3389/fninf.2020.00027

**Published:** 2020-09-11

**Authors:** Petr Ježek, Jeffery L. Teeters, Friedrich T. Sommer

**Affiliations:** ^1^Faculty of Applied Sciences, New Technologies for the Information Society, University of West Bohemia, Plzeň, Czechia; ^2^Redwood Center for Theoretical Neuroscience & Helen Wills Neuroscience Institute, University of California, Berkeley, Berkeley, CA, United States

**Keywords:** NWB format, HDF5, neurophysiology, metadata, search, Java, Python, SQLite

## Abstract

The Neurodata Without Borders (abbreviation NWB) format is a current technology for storing neurophysiology data along with the associated metadata. Data stored in the format is organized into separate HDF5 files, each file usually storing the data associated with a single recording session. While the NWB format provides a structured method for storing data, so far there have not been tools which enable searching a collection of NWB files in order to find data of interest for a particular purpose. We describe here three tools to enable searching NWB files. The tools have different features making each of them most useful for a particular task. The first tool, called the NWB Query Engine, is written in Java. It allows searching the complete content of NWB files. It was designed for the first version of NWB (NWB 1) and supports most (but not all) features of the most recent version (NWB 2). For some searches, it is the fastest tool. The second tool, called “search_nwb” is written in Python and also allow searching the complete contents of NWB files. It works with both NWB 1 and NWB 2, as does the third tool. The third tool, called “nwbindexer” enables searching a collection of NWB files using a two-step process. In the first step, a utility is run which creates an SQLite database containing the metadata in a collection of NWB files. This database is then searched in the second step, using another utility. Once the index is built, this two-step processes allows faster searches than are done by the other tools, but does not enable as complete of searches. All three tools use a simple query language which was developed for this project. Software integrating the three tools into a web-interface is provided which enables searching NWB files by submitting a web form.

## 1. Introduction

Effective management of neurophysiology data requires not only storing the data on disk or in some other medium, but also having a method in place to enable efficient search for finding parts in the data that are needed for some purpose. The efficiency of methods of data search depend on how the data are stored. If the data are stored so that all components of the data are accessible using a single software tool, then the searches can be performed using that software. For example, the DataJoint software (Yatsenko et al., 2015) uses a relational database to store data, and if all data needed to be searched was stored within a single DataJoint database, then searches could be done using tools designed for searching relational databases, such as SQL.

However, for various reasons (including: convenience, a potentially large size of the data, ease of data exchange, compatibility with software tools), currently neurophysiology data are often not stored as a single integrated collection such as a relational database, but instead, data are stored in multiple, independent files. These files are typically organized by experimental sessions, so that data recorded in separate sessions of an experiment are stored in separate files. Metadata are either stored in these files or in an external file typically in JSON or XML format. There are many file formats used to store neurophysiology data in this manner. Examples are: the BrainVision data format^1^ used by Brain Products Analyzer; the European Data Format (EDF) (Kemp and Olivan, 2003); standard ASCII used by EEGLab (Delorme and Makeig, 2004); the BDF format, a variation of EDF used in BioSemi^2^ products; Spike2 format (Smith, 2003); Klustakwik (Harris et al., 2000; Rossant et al., 2016); NIX (Stoewer et al., 2014); BIDS (Gorgolewski et al., 2016); NSDF (Ray et al., 2016); and Sonata (Dai et al., 2020).

Another recently developed format that stores data in individual files is the Neurodata Without Borders (or NWB) format (Teeters et al., 2015; Rübel et al., 2017, 2019). The NWB format stores different types of neurophysiology data in a standard manner to allow data to be more easily shared across labs and to potentially make data easier to use within a laboratory. The NWB data format is open source, includes a Python and MATLAB API, and uses HDF5 as a backend. The NWB format is becoming well-established in the neuroscience community as evident by the fact that: its development has been funded by the National Institutes of Health (NIH); hackathons, tutorials, and workshops about the format are being held each year; it has been adopted by labs in the Allen Institute for Brain Science^3^ and other major labs, and the format won a 2019 R&D100 Award^4^.

Searching data that is distributed across many individual files, such as the aforementioned formats, requires software that is customized to read the data in the individual files and provide some type of interface that allows searching it. Here, we present such software for the NWB format—a suite of NWB query engines. The tools allow searching data within NWB files that are stored locally and also (using the third tool) searching contents of files that are not local after an index of the file contents is constructed. We also provide a web interface to all of the tools, which enables searching files (or index of file contents) that are stored on remote systems running the query engine. This enables search in files without having to first download the files. This feature is useful if the files are large, which is often the case with neurophysiology data.

To make the query tools as easy-to-use as possible, and to accommodate the hierarchical organization of data within the NWB format, the specification of the search in each tool (that is, the query), is done using a custom query language that was developed for this project and is simpler than SQL. Although the main motivation for this project was to provide a method for searching NWB data, the solutions we provide could also be useful for querying data in other formats.

The paper is organized as follows: section 2 describes some related software and the requirements for the search tools. Section 3 describes the developed tools. Lastly, section 4 summarizes the tools and assumptions made when developing them and describes how they may be used and extended.

## 2. Materials and Methods

### 2.1. The HDF5 Data Model

The NWB format uses HDF5. HDF5 is a technology suite that includes a data model, library, and file format for storing and managing data (Folk et al., 2011; Koziol, 2011). HDF5 is used in many science disciplines and software supporting it is available on all major platforms^5^. In addition to NWB, several other neuroscience software systems use HDF5, including: NIX Stoewer et al. (2014), which provides a domain-independent way of storing data—and associated metadata using odML (Grewe et al., 2011); the KlustaKwik tools used for spike sorting (Rossant et al., 2016); and the BRAINformat (Rübel et al., 2016).

The HDF5 data model contains three main entities:

- **Groups** are containers that can contain other groups and datasets.
- **Datasets** are multidimensional arrays of data. The data can be various types, including built-in types (such as integers, strings, floating point numbers and so on), or user-defined types that are composites of the built-in types.
- **Attributes** are a key-value dictionary associated with a group or dataset. They map a key (an attribute name, which is a string) to a value which can be a scalar value or an array.

The groups and datasets are used to create a hierarchical organization of data within an HDF5 file, analogous to the organization of files within directories on a file system. In this analogy, groups correspond to directories in a file system and HDF5 datasets correspond to files within the directories. The sequence of groups (starting from the top, or root group) to a group or dataset in an HDF5 file is known as the “absolute path” or “location”. The attributes are used to store metadata associated with groups and datasets.

In addition to the entities listed above, the HDF5 format also allows links between components within a file and between files. These are analogous to hard and soft links in a file system. The links between files (called external links) allow data to be stored in multiple files, but accessed through the HDF5 API as a single hierarchical structure.

### 2.2. The Neurodata Without Borders Format

The NWB format was created to provide a standard way of storing neurophysiology data. The NWB format is currently available in two versions, NWB 1 (also called NWB:Cellular Neurophysiology or NWB:CN) (Teeters et al., 2015) and NWB 2 (also called NWB:Neurophysiology or NWB:N) (Rübel et al., 2017, 2019)^6^. This section summarizes the features of both versions that are important for searching data stored using the format.

#### 2.2.1. Overall Layout

The experimental data typically stored in NWB files consists of data that is invariant throughout the session (such as information about the subject, anatomical locations of the electrodes, and so on) and data that varies throughout the session (such as measurements of neuronal activity and behavior, stimuli, and processed data).

**Figure 1:**
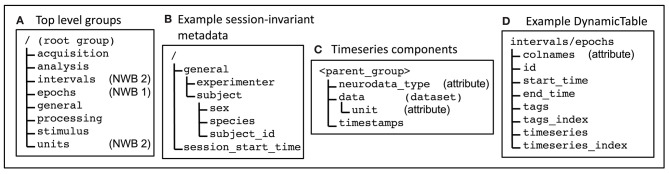
Some of the main data layouts the NWB format. **(A)** Top-level groups are used to broadly organize the data. **(B)** Some commonly used session-invariant metadata. In **(A,B)**, the leading slash in the location indicates that these are at a fixed location in the HDF5 file (full absolute path is specified). **(C)** NWB timeseries are used to store data that varies with time. For each timeseries, the components are all stored within a parent group that has a variable name (chosen by the creator of the file) and can be located in different places within the NWB file. **(D)** The NWB DynamicTable layout stores data that is logically organized as a table in multiple datasets. Multiple values in the same cell (non-scalar values) are supported using pairs of datasets: one to store the values, e.g., tags; and the other to store value indices, e.g., tags_index.

To store these different data types in a consistent manner within HDF5, the NWB format prescribes a standard layout (organization of HDF5 groups, datasets and attributes) for each type of data. Some commonly used layouts are shown in Figure 1. Most of the HDF5 groups and datasets specified in the format have a fixed location (all components of the absolute path are specified). These include the top-level groups (Figure 1A) and session-invariant metadata (Figure 1B). The format also uses HDF5 groups and datasets that do not have a fixed location, but instead some components of the path are “variable” (that is chosen during creation of the NWB file). Variable paths allow multiple instances of a layout to be stored in the same file (but in different locations). An examples is the TimeSeries layout (Figure 1C), which are stored in many different top-level groups within an NWB file. NWB version 2 provides a layout called “DynamicTable” for storing data that are organized into tables. An example is the content of the /interval/epochs group (Figure 1D). The DynamicTable layout is described in section 2.2.2.

In addition to the built-in layouts summarized above, the NWB format can be extended to include additional HDF5 groups and datasets in order to store types of data that are not described by the built-in layouts. These may also be in variable locations. The built-in layouts and any extended layouts are usually stored within a single NWB (HDF5) file, but sometimes the data are split between multiple files and HDF5 external links (described in section 2.1) are used to maintain the relationship specified by the layouts even though the contents are in multiple files.

#### 2.2.2. Representation of Tables

There are many instances in which tabular data (that is, data organized similar to a spreadsheet, where columns indicate different features, and rows indicate instances of an item having the features) must be stored in NWB files. Examples of the kinds of data and the features stored in tables are: *experimental epochs* (features are start time, stop time, trial Id, tags indicating trial properties), *electrode locations* (features are x, y and z coordinates in some reference frame), properties of individual neurons (*units*) (some features are: unit id, location in brain, spike waveform, spike time, quality of recording).

The storage of tabular data within HDF5 requires different methods depending on the characteristics of the data. If all the columns in the tabular data contain only a single scalar value (e.g., an integer or real number or a string^7^) and if the type of data in all columns are the same (e.g., all columns store integers), then the table can be stored directly in HDF5 using a single HDF5 dataset containing one of the built-in HDF5 data types. However, if either some of the cells can contain multiple values or if the type of data in the table is heterogeneous (for example, one feature is integer, another string), then a single HDF5 dataset that contains elements of the HDF5 built-in types cannot be used to store the table since such HDF5 datasets store only arrays of homogeneous values. The methods used in the NWB format to store tabular data in these two cases (non-homogeneous data types and non-scalar values) are described below.

##### 2.2.2.1. Storing non-homogeneous tabular data

**Figure 2:**
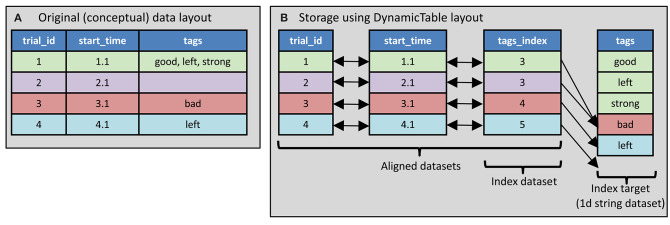
Table storage using the DynamicTable layout. **(A)** The table to store contains three columns: trial_id (an integer), start_time (a float) and tags (zero or more strings). Trial_id 1 (first row) has three tags, trial_id 2 has no tags, and the other two trials each have one tag. **(B)** Storage in NWB format using the DynamicTable layout. The tags column is stored using two datasets: tag_index and tags. The tags dataset contains all the tags from all trials concatenated. The trial_id and start_time and tags_index are store in separate datasets with their elements aligned according to each row. (This illustrates aligned datasets). The tags_index array indicates which tags (elements of the tags array) are in each row of tags column of the table. Each element of tags_index is the index just beyond the last value included for that row. (This illustrates the index array).

**Figure 3:**
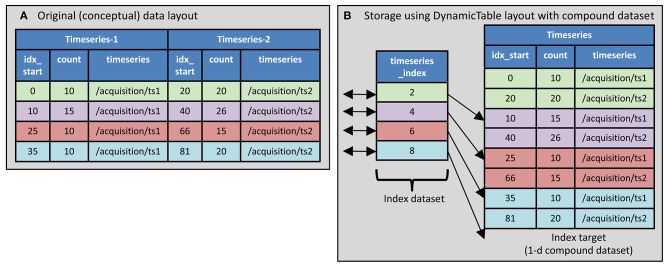
Compound datatype used in DynamicTable layout. **(A)** The table to store contains information indicating what part of two different timeseries data layouts correspond to the trial intervals. There is information about two timeseries. **(B)** The HDF5 compound datatype is used along with an index array to indicate which timeseries segments are associate with each trial.

To store tabular data that contains non-homogeneous data types, two methods are used. These are:

- Use *Aligned datasets*. The tabular data is stored using multiple datasets, each of which contains data of a single type. These tables are aligned on the first dimension. For example, a table that contains an integer in one column and a float in another can be stored as two datasets, one containing the integer column and the other containing the float column. To access the contents of a row, the corresponding element from both dataset are retrieved. This is illustrated in Figure 2B. In Rübel, 2019, this method is referred to as “column-based” tables. This method is used in both NWB 1 and NWB 2 although in NWB 1 (unlike NWB 2) there is no indication at the level of the enclosing HDF5 group that the contained datasets are aligned. In NWB 2, the “DynamicTable” layout is used to store such data. This layout uses an attribute named “colnames” on the enclosing group that list the datasets within the group storing columns of the table and each column may have an associated index array which is used for storing non-scalar values. The index array is described in section 2.2.2.2.
- Use a *compound data type*. With this method, a HDF5 “compound” data type is defined that includes all of the data fields, then a dataset is created which has elements of that compound type. This method is used only in NWB 2. An example is shown in Figure 3.

##### 2.2.2.2. Storing tabular data with non-scalar elements

To store tabular data that contains non-scalar values in one or more columns, there are also two methods used. These are:

- Use a *Separate group for each row*. Each row in the table is stored in a separate HDF5 group that is named to indicate the order of the row in the table, e.g., “epoch_001.” Within each group the columns that have scalar values are stored as a dataset containing only the scalar value. The columns that have multiple values are stored as a dataset containing the multiple values. In Rübel et al. (2019), this is referred to as “Implicit Ragged Arrays.” This method is only used in NWB 1.
- Use an *index array*. The columns that may contain multiple values are stored using two datasets: a single “value” dataset that contains all the values from all rows concatenated together, and an “index” dataset that contains for each row, the index of the element in the value dataset after the last element that goes into that row. Through this, the index dataset specifies which elements in the value dataset are associated with a given row of the table. It is used in the DynamicTable layout in NWB 2 (see Figures 2, 3). This method is called “Region-based Ragged Arrays” in Rübel et al. (2019). It is used in NWB 2 only.

### 2.3. Required Searches

The NWB Query engine tools were developed to enable searching multiple NWB files in order to find those that match a search criterion and to also search an individual file to find parts of the file matching a search criterion. For both purposes, the search criterion is specified as one or more entities within the HDF5 file and a characteristic each entity must satisfy. Since the NWB format includes layouts that have both a fixed and variable location (as described in section 2.2.1), it must be possible to search for criteria for entities stored at both types of locations. It also must be possible to search for tabular data that is stored using the different methods described in section 2.2.2 and search data that is organized into multiple files using HDF5 external links as described in section 2.2.1.

### 2.4. Existing Search Tools for HDF5

HDF5 does not natively include a search capability, but several tools to search HDF5 files have been created. HDF5-FastQuery (Gosink et al., 2006; Chou et al., 2011) provides a fast way of finding subsections of multi-dimensional data in HDF5 datasets by creating bitmap indices of datasets that are stored within the original file. Another indexing approach for HDF5 (Wang et al., 2013) creates a light-weight data management layer that allows running queries expressed in the SQL syntax on a metadata storage which is generated from an HDF5 file at runtime. A third tool, HDFql^8^ also facilitates working with HDF5 files by providing a SQL-like language interface.

These tools cannot be used to perform the required searches (section 2.3) because all of them (except for HDFql) require that the full location of the HDF5 entities being searched for are known in advance and none of them allow searching a collection of files with one query. Furthermore, none of the tools allow searching tabular data stored using all of the methods described in section 2.2.2.

### 2.5. Scope and Requirements

The NWB Query Engine tools must implement the required searches described in section 2.3. Basic relational and set operators must be provided to support comparisons and union and intersection of partial results. The implementation should not narrowly focus on just the NWB format, but should be extensible to other formats. Since many tools in neuroscience are implemented in Python (Muller et al., 2015), a Python interface must be available. To make the system easy to use (e.g., without requiring the installation of local software or requiring learning a complex query language) the system must be accessible using a web interface and the queries must be easy to specify, e.g. easier than SQL.

## 3. Results

### 3.1. NWB Query Engine Tools

Guided by the requirements given in section 2.5, we created a suite of software tools that facilitate querying NWB files. The software suite consists of three independent query systems that work in complementary ways. The first, called the “NWB Query Engine,” allows performing a query on one or more NWB files by searching within the HDF5 files directly. This software is written in Java and can be called from both Java and Python. It was designed for NWB 1, but can also be used for many types of searches of NWB 2 files. The second query system, called “search_nwb” is written in Python. Like the NWB Query Engine, it enables searching one of more NWB files by reading them directly, but unlike the NWB Query Engine, it allows searching for data stored using all of the table representation methods described in section 2.2.2. The third tool, called “nwbindexer” is also written in Python. It works by first creating an SQLite^9^ index of content in one or more NWB files, then allows searches to be performed on the index. Once the index is built, this enables faster searches than the other two methods. It allows searching all of the table representations described in section 2.2.2, but does not allow searching the entire contents of an NWB file because only a subset of the file is stored in the index.

All three of these software tools use the same language to express queries. This language is described in the next section, followed by a description of each of the query systems.

### 3.2. Query Grammar

To allow performing the required searches described in section 2.3, an easy-to-use query language was developed. This language allows a user to define a query using subqueries. Each subquery consists of two parts separated by a “:”. The left side is the name of a “parent” group or dataset. The name consists of the HDF5 path to the parent and may include one or more asterisk “*” (wildcard character) which will match any sequence of characters. The right side is an expression containing the names of “child” items to search for within the parent along with value restrictions. These items will be attributes of the parent if the parent is a dataset, and they will be attributes or datasets if the parent is a group. A query can consist of just one subquery or be composed of multiple subqueries that are joined by logical and (“&”) and or (“|”). The composite query succeeds (finds items matching the query specification) if the logical expression formed with the subqueries is True when the subqueries that are successful are evaluated as True and the subqueries that do not succeed are evaluated as False.

The following operators are supported to define value restrictions for the items (attributes or datasets) named on the right side of the colon:

- and **&**
- or **|**
- parenthesis **()**
- substrings in strings **LIKE**
- relational operators: **<** lesser than, ** <=** lesser or equal, **>** greater than, **>=** greater or equal, and **==** equal.

**Figure 4:**
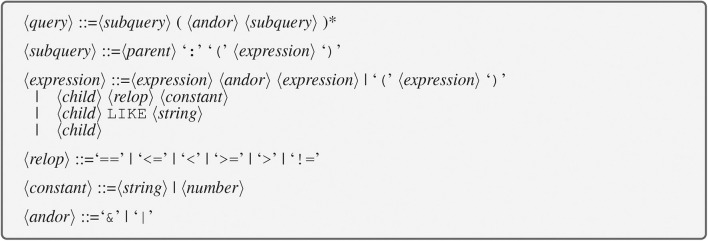
Formal description of query grammar in BNF form. <*query>* is made up of one or more subqueries. The ()* construct at the end of <*query>* indicates zero or more occurrences. <*parent>* is a path to an HDF5 group or dataset. <*child>* is the name of an attribute or dataset within the parent. <*string>* is a string constant enclosed in single or double quotes. <*number>* is a numeric constant.

A formal description of the grammar is given in Figure 4. Some sample queries are shown in Table 1. The grammar is simpler than the grammar of SQL SELECT statements (which has keywords, table alias, joins, and so on) because SQL is designed for relational databases which use tables with primary and foreign keys to organize data whereas the grammar defined here is intended to operate on the hierarchical structure of HDF5 files, which is more straightforward.

**Table 1:** Query Examples.

**Query**	**Description**
/general/subject: (species == "Mus musculus")	selects all files with the specified species.
/general:(virus)	selects all records with a virus dataset
/general:(virus LIKE "%infectionLocation: M2%")	selects all datasets virus with infectionLocation: M2
*:(neurodata_type == "RoiResponseSeries")	select all TimeSeries containing Calcium imaging data
*/data: (unit == "unknown")	selects all datasetes data which unit is unknown
*/epochs/*: (start_time > 500 & start_time < 550 & tags LIKE "%HitL%" & tags LIKE "%LickEarly%")	select all epochs with the matching start_time and tags
/general/subject: (subject_id == "anm00210863") & */epochs/*: (start_time > 500 & start_time < 550 & tags LIKE "%LickEarly%")	select files with the specified subject_id and epochs
/units: (id > -1 & location == "CA3" & quality > 0.8)	select unit id where location is CA3 and quality > 0.8

*The left column shows query examples. The right column describes the expected result*.

### 3.3. NWB Query Engine

In this section we describe the NWB Query Engine, which is the first of the three query systems.

#### 3.3.1. Architecture

**Figure 5:**
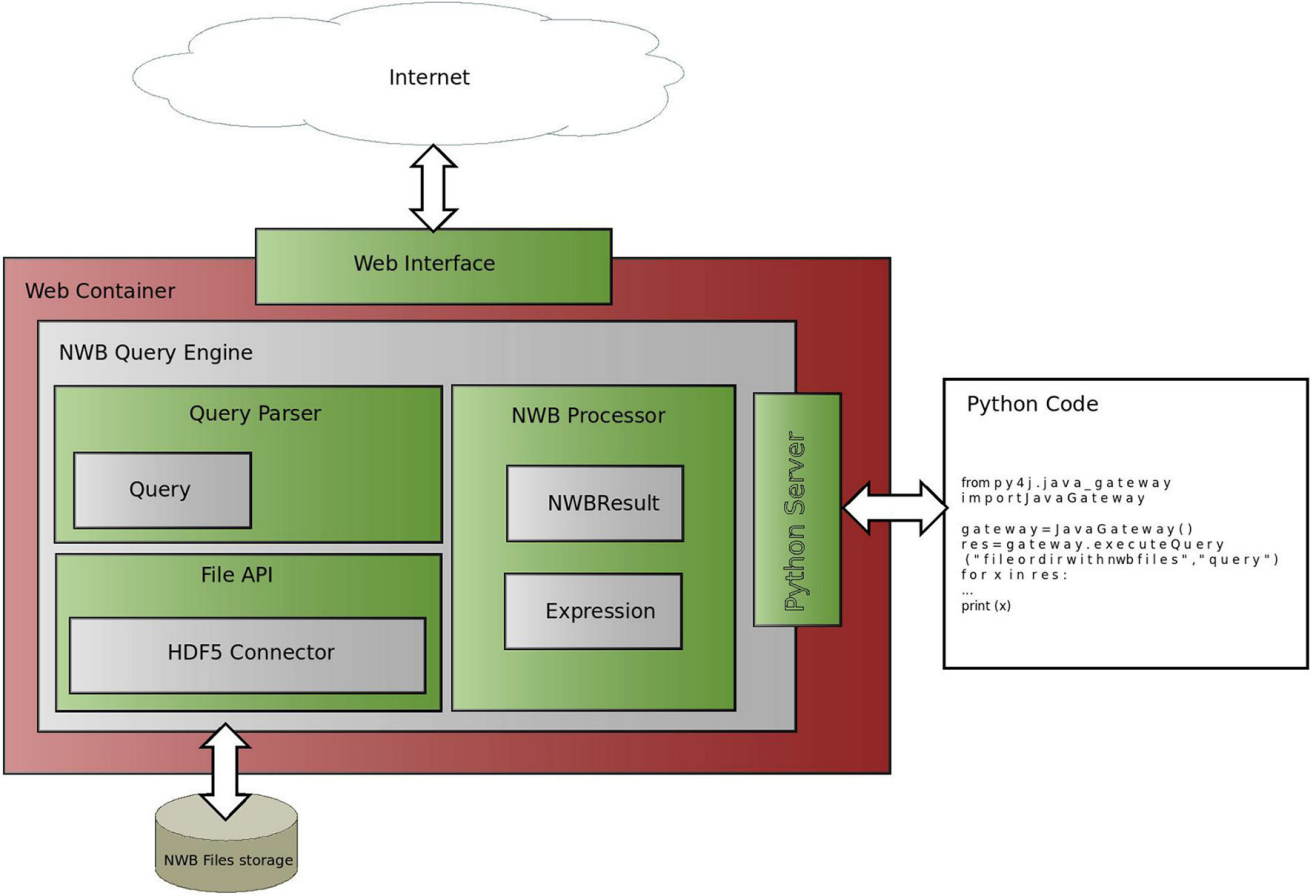
Architecture of NWB Query Engine. The NWB Query Engine is composed of three core components: the Query Parser, the File API and the NWB Processor. The query parser translates a user input into an internal tree representation. The NWB Processor uses the tree to perform searches in a NWB file through the HDF5 Connector. The retrieved data are wrapped in a NWBResult object, which is returned by the query engine. The individual blocks communicate via interfaces to facilitate using the software in different environments, for example, using different data storage methods. The Python server enables calling the query engine from Python code. The Web Interface provides a user-friendly web page that allows running queries from a web browser. The Web Container is a part of the web server that processes user requests and communicates with the NWB Query Engine.

The architecture of the NWB Query Engine is shown in Figure 5. The NWB Query Engine contains three main components: the Query Parser, the NWB Processor, and the File API. The first component, the Query Parser, parses a query that is expressed according to the grammar described in section 3.2 and unfolds it into a binary tree that has an operator at each internal node and operands at the leaves. The binary tree is used by the second component, the NWB Processor. The NWB Processor processes the tree by searching within the NWB file for HDF5 attributes or datasets that fulfill the constraints specified by the expression encoded in the tree. The search within the NWB file is performed by the HDF5 connector, which is part of the third component, the File API. Each dataset that satisfies the search criteria is used by the NWB Processor to create an NWB Result object, which contains the location of the NWB file, the path within the NWB file to the dataset or attribute and the matching value. Since the File API performs all searches within NWB files, this one component can be updated to allow the Query Engine to work with different NWB storage methods (other than HDF5) or different data formats.

The software interfacing to the NWB Query engine includes the Python server and Web Interface. The Python server can run on a local or a remote host and enables processing queries using Python code. The Web interface is described in section 3.7.

#### 3.3.2. Operation and Limitations

As part of the step of evaluating expressions, the NWB Query Engine compares the value of each dataset or attribute referenced in the query to the constant given on the right hand side of the relational operator independently of any other datasets or attributes referenced in the query. If the value is a scalar (which is often the case for metadata) the comparison is done on the retrieved scalar value. For example, if the query has the expression “x > 4” and the value of x retrieved from the NWB file is the scalar 5, then the expression would be evaluated as True. If the value retrieved is not a scalar (e.g., has multiple elements) then the comparison is done on each independently to find all elements that evaluate as True. For example, if the value retrieved by x was a dataset containing three elements, (6, 4, 5), then with expression “x > 4” the Query Engine would evaluate True for the first and third since these elements are greater than 4, but not the second.

One consequence of this method of evaluation is that the NWB Query Engine cannot be used to search tabular data stored using the aligned datasets or the index array methods (described in section 2.2.2) that satisfy more than one constraint since it will return results for each constraint independently regardless of whether or not the elements are in the same row. For example, if the query is: “quality > 0.95 & location LIKE "CA1"” and both quality and location are columns in a table stored using the aligned datasets method, then the NWB Query Engine will return a matching result if any value in the quality and location dataset satisfy these conditions, rather than returning a match only if values in the same row satisfy the condition. Other limitations are that the NWB Query Engine cannot currently search compound datasets or HDF5 Object references^10^ (which are also used in NWB 2). This is because it is built on top of the HDFql library which cannot currently read HDF5 compound data types or object references. These limitations arose because the NWB Query Engine was developed before NWB 2 was available and it was not designed to query data stored using the DynamicTable layout used in NWB 2. The details of the implementation of the NWB Query Engine are given in the Supplementary Materials.

### 3.4. search_nwb

The search_nwb utility was developed to overcome the limitations of the NWB Query Engine for searches of NWB 2 files. In particular, in addition to searching layouts common to NWB Versions 1 and 2, it also allows searching tabular data stored using the DynamicTable layout described in section 2.2.2 and also searching HDF5 object references^11^ which are used in NWB 2 but not NWB 1. Like the NWB Query engine, the search_nwb utility works by directly reading NWB files and searching for contents that match the specified query. It is written in Python and uses the H5py library^12^ to read the contents of the NWB files.

#### 3.4.1. Enhancements to Query Grammar

Both search_nwb and nwbindexer (described in the next section) accept queries that conform to the grammar given in section 3.2. However, both of these utilities also support some extensions to the grammar. These extensions are:

1. The parentheses around the subquery part on the right of the colon are optional.
2. Each subquery can include a list of children of the parent immediately after the “:”. The values of these children are included with the results even if they are not used in an expression that specifies constraints on the values. The elements in the list can be separated by a single comma and/or spaces.
3. To allow searching compound datasets and two-dimensional datasets that are used within a DynamicTable layout, a column index of a 2-d dataset or a component name of a compound dataset may be specified inside square brackets after the child names used in a query.

The following example query illustrates these extensions:

   intervals/epochs: id, tags, start_time, stop_time, timeseries[timeseries] LIKE ~%test%~

In the above example: There is no parentheses around the query part to the right of the colon. The variables “id” “tags,” “start_time,” and “stop_time” are the names of children of the parent for which the values are included in the search results. The string “timeseries” appears twice. The first occurrence (before the square brackets) is the name of the compound dataset, and the second occurrence (within the square brackets) is the name of a component of the compound dataset. (In general, these will not be the same, but they are in this example because that is how the data is organized.) The layout of the compound dataset being searched is illustrated in Figure 3B. It is in the interval/epochs group as illustrated in Figure 1D.

#### 3.4.2. Implementation

The implementation of the search_nwb utility leverages the Python “eval” function which allows executing strings that contain Python code from within a Python program and also the Python “filter” function which enables performing operations on corresponding elements of aligned lists. The “filter” function is used to find which values satisfy the query expression. Details of the implementation of the search_nwb utility are in section 2 (Supplementary Materials).

### 3.5. nwbindexer

Because both the NWB Query Engine and search_nwb tools work by reading though NWB files to process each query, they can become slow to complete a query if there is a large number of files or HDF5 nodes that need to be searched. In order to speed up the search process, nwbindexer was developed. Unlike the other NWB Query Engine tools, nwbindexer operates in two steps. First, a program (called *build_index.py*) is run, which reads the contents of NWB files and stores a subset of the contents into a SQLite database (also called the “index”). This database is used by the second program, called *query_index.py*, that allows searching the SQLite database using queries that are in the same form as used in the search_nwb tool. These programs and the database are described below.

#### 3.5.1. *build_index.py*

The *build_index.py* program has a required command line parameter which is the path to a directory containing NWB files. It scans the directory for NWB files and for each NWB file found, it adds a subset of the content to the SQLite database. The subset of content added is described in section 3.5.3. The *build_index.py* program creates the SQLite database, and there is no additional action needed by the user to setup or administer the database.

#### 3.5.2. SQLite Database Schema

**Figure 6:**
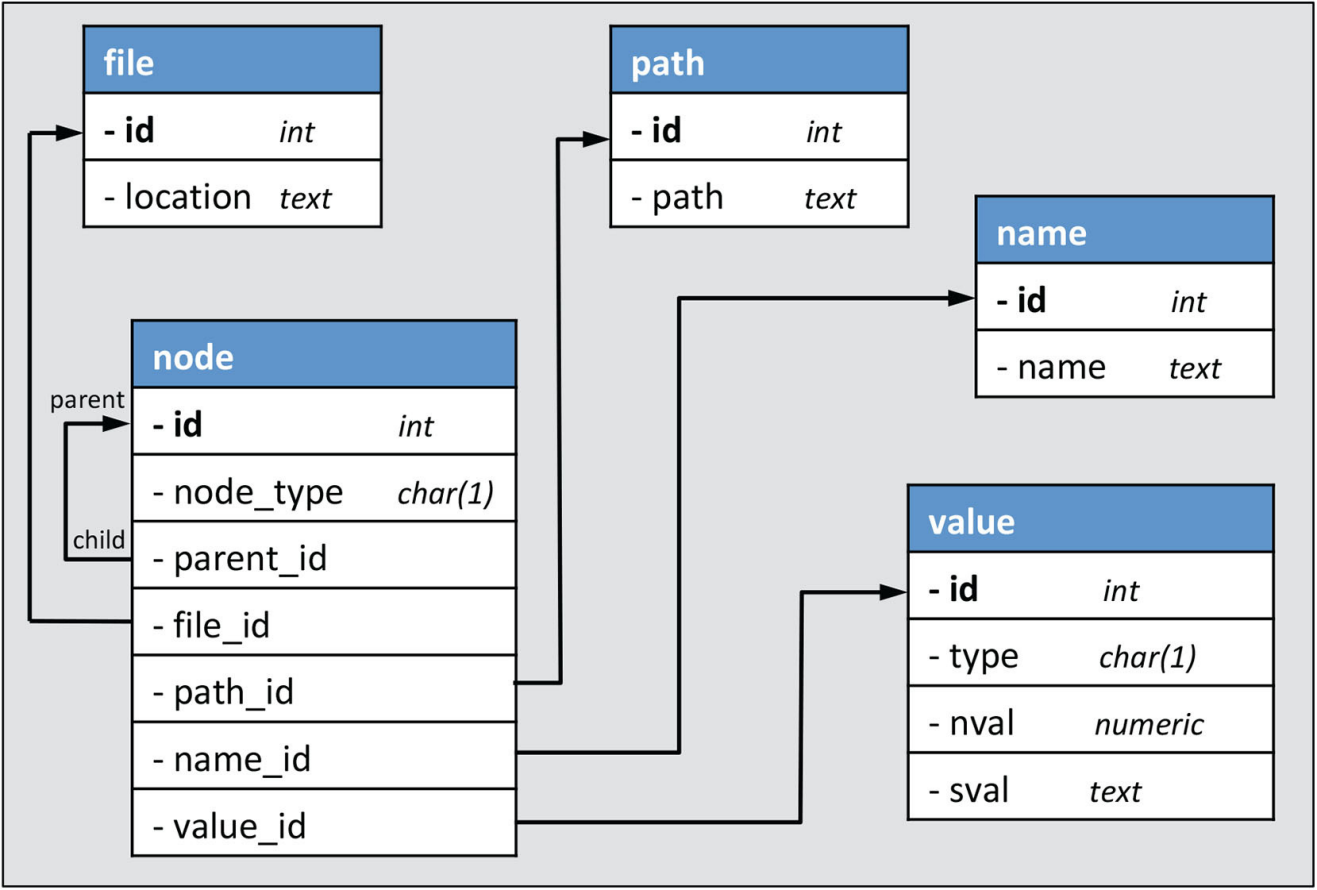
SQLite database schema used for nwbindexer. Each arrow indicates a 1:M (one-to-many) relationship from a foreign key to a primary key.

The schema that is used to store the subset of the NWB file contents is shown in Figure 6. There are five tables. The names of the tables and a summary of the contents are as follows:

- **File** - contains the location (full path on the file system) of every NWB file which is indexed (has values stored in the database).
- **Path** - stores the path to all nodes in an HDF5 file that have children (either attributes, groups or datasets). For example, the path “a/b/c” would be stored in the path table if node “c” had a child, otherwise path “a/b” would be stored (since “b” has at least one child, that is “c”).
- **Name** - stores the last component of the HDF5 path to each node. This allows the full path to a child to be formed by appending the name of the node to the path associated with the parent node.
- **Node** - contains information about each HDF5 node, where node (as used here) includes not only groups and datasets, but also attributes.
- **Value** - contains values of both datasets and attributes.

**Figure 7:**
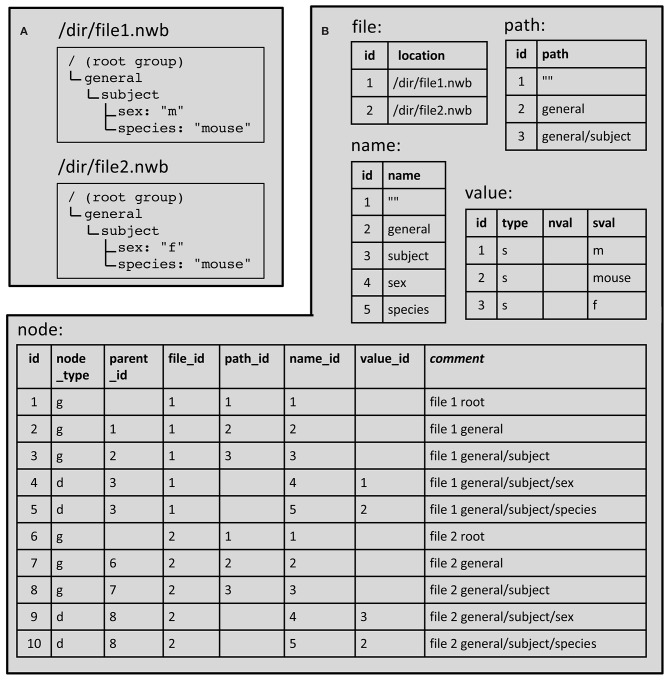
Example NWB file hierarchy stored using SQLite database tables. **(A)** NWB file contents. **(B)** Corresponding database tables. Table entries that are empty store a NULL. The node table field “node_type” is “g” for group and “d” for dataset. The value table “type” field is “s” to indicate a scalar string. Details are given in sections 3.1 and 3.2 (Supplementary Materials).

An example showing how these tables are used to represent hierarchically organized content in two NWB files is given in Figure 7. Of the five tables in the database, only two of them (“file” and “node”) contain contents that are not shared across files. In other words, there is a unique row in the file table for every file and a unique row in the node table for every group, dataset and attribute in every file. The other tables (path, name, and value) store content that is shared across files. This reduces the space required to store the information because a given path, name or value is only stored once regardless of how often it may be used in all the files. It also facilitates efficient searches since it allows using one SQL query to find all files that satisfy a particular path or value constraint.

#### 3.5.3. Storing Values

Data values contained within NWB files (that is, values of HDF5 attributes and datasets) are stored in the database “value” table. The value table “nval” field stores only a single numeric value (integer or float). The “sval” field is used to store all other types of data including strings, string arrays, and numeric arrays that are in a DynamicTable. The Python csv^13^ module is used to encode arrays as comma separated values for storing in the sval field. All strings (if they are not already in Unicode) are converted to Unicode before saving in the value table. All object references are first converted to the path (a string) of the object being referenced.

When constructing the database, a judicious selection of what values to store must be made because it is impossible to store all values (otherwise the database would be too large since it would include the full contents of all indexed NWB files). Since the database is used for searching, only values that are likely to be referenced in searches should be included. To do this, two sets of criteria were used:

First, for values that are not part of a DynamicTable, only scalar numeric values, scalar strings and short string arrays (up to 20 elements long) with a total length (of string or string arrays) not greater than 3000 characters are saved^14^. This is because these types of values are likely to be metadata referenced in searches^15^.

Second, for values that are part of a DynamicTable (that is, in a DynamicTable column), all values are saved (up to a cutoff length, currently 10,000 elements per column) because these are also likely to be referenced in searches. Details of the storage of values are given in section 3.2 (Supplementary Materials).

#### 3.5.4. *query_index.py*

The *query_index.py* program has a required command-line argument which specifies a database (built by *build_index.py*) to query. It can either process a query entered on the command line or process queries entered interactively.

Like the search_nwb tool, the *query_index.py* utility uses the Python eval and filter functions to find values which match the constraints specified by the expression within each subquery. However, the steps used in the *query_index.py* utility are different from those used in search_nwb because, to find the groups or datasets to check for matches to the expression, the search_nwb utility must read the NWB files directly, whereas the *query_index.py* program does this by querying the SQLite database. Details of the implementation of nwbindexer are described in section 3 (Supplementary Materials).

### 3.6. Performance

A variety of queries were used to test the performance of the tools. The queries included comparing two numeric values, searching for exact string, searching a substring using the LIKE construct, searching values stored in a DynamicTable and searching using wildcards in the parent path. Compound queries with logical operators were also tested.

The tests were performed on a collection of 70 NWB files with a total size of 31GB. Each search was run 12 times. The average time, as well as the minimum and maximum time, needed to execute each search was recorded over each query and tool. The time required to build the index used by nwbindexer (that is the time required to run the *build_index.py* program, which took about 8 minutes) was not included in the time for nwbindexer because it only needs to be done once.

The NWB files used for testing are listed below. The first item in the list are NWB 1 files; all others are NWB 2. Except for the first and last items in the list, all files in the list are referenced on the Examples page on the NWB website^16^.

- The first 16 files from the alm-1 data set at CRCNS.org, which contains anterior motor cortex recordings from the Svoboda Lab at Janelia Farm^17^ (2.2 GB).
- A file from a data set referenced in a Nature publication (Steinmetz et al., 2019) that was converted to the NWB format^18^ (267.96 MB).
- A data file from the Buzsáki Lab; File: YutaMouse41-150903.nwb^19^ (10 GB).
- Files in file “nwb_1_28.zip” from the Anne K Churchland lab^20^ (28 files, 17 GB total).
- Files generated from the PyNWB tutorials^21^ in commit fcb919a3^22^ (22 files, 542 MB total).

**Figure 8:**
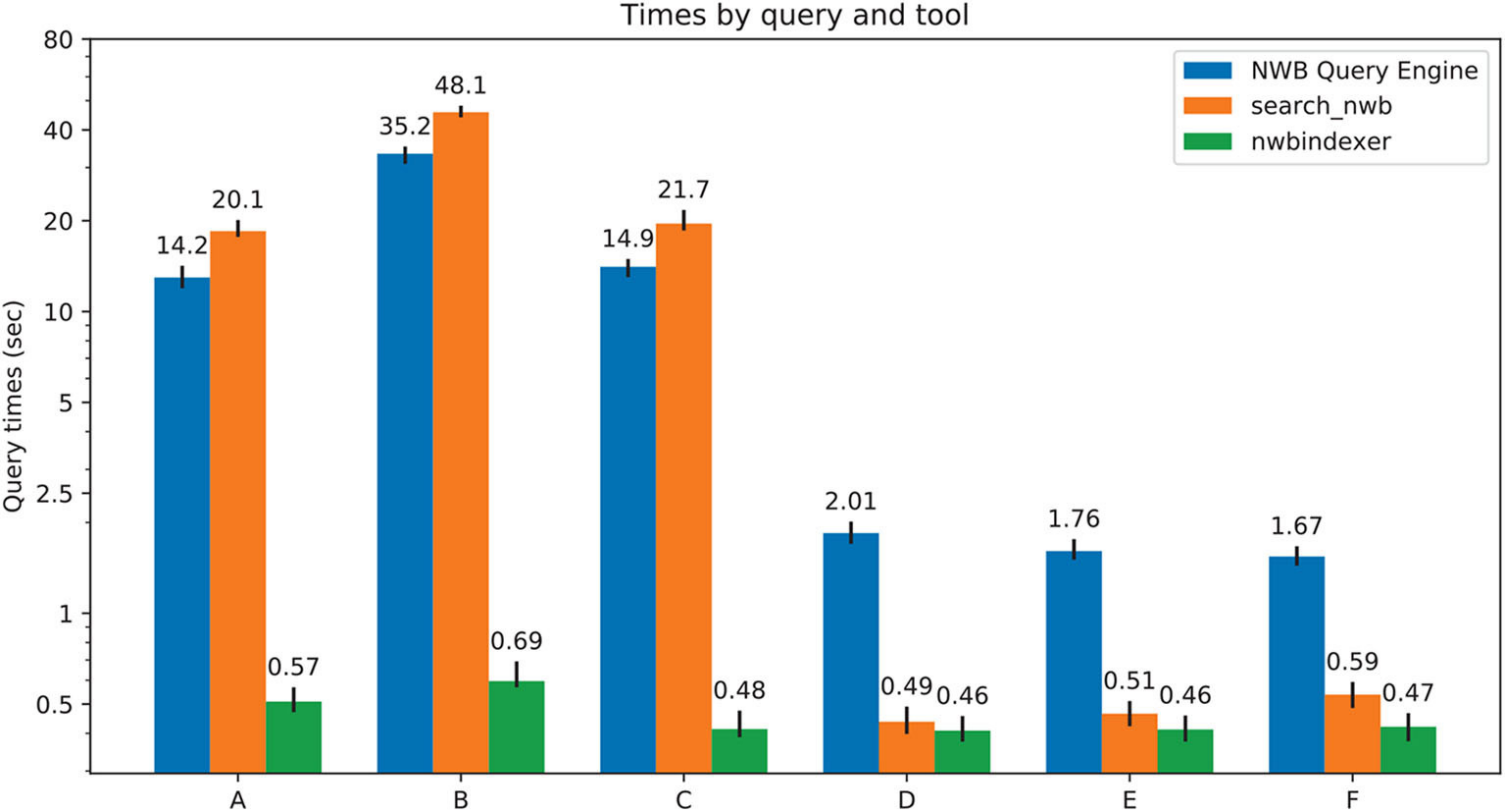
Comparison of performance of all the three tools. The numbers above each colored bar are the average time for the query using that tool. The vertical line passing through the top of each bar shows the range between the minimum and maximum times. The tested queries are: **(A)** epochs*:(start_time > 200 & stop_time<250 | stop_time>4850), **(B)** */data: (unit == "unknown"), **(C)** general/subject: (subject_id == "anm00210863") & epochs/*: (start_time > 500 & start_time < 550 & tags LIKE "%LickEarly%"), **(D)** units: (id > -1 & location == "CA3" & quality > 0.8), **(E)** /general:(virus LIKE "%infectionLocation: M2%"). **(F)** general/optophysiology/*: (excitation_lambda).

The test results are summarized in Figure 8. For Figure 8 queries A-C, nwbindexer was over 20x faster than the other two tools, and the NWB Query Engine was about 1.5x faster than the search_nwb tool. These queries have wildcards in the parent location that match lots of groups in the HDF5 hierarchy. The nwbindexer tool is much faster for such queries because the matching groups are found using a SQL SELECT statement, whereas the other two tools sequentially search for the many matching groups and that negatively affects their performance.

For Figure 8 queries D-F, nwbindexer and search_nwb were about the same speed, with nwbindexer slightly faster. Both of these tools were about 3x to 4x faster than the NWB Query Engine. These queries included: comparisons of scalar values, LIKE conditions with wildcards in the matching pattern and compound queries.

These results indicate that for data that is indexed by nwbindexer, it would probably be the fastest tool. However, as mentioned in section 3.5.3, not all data in an NWB file is indexed by nwbindexer. For searching for data that is not indexed by NWB indexer, in some cases (queries that reference lots of HDF5 nodes and for data not stored using the DynamicTable layout) the NWB Query Engine will likely be the fastest tool. For other cases (queries matching fewer nodes or which search DynamicTable layouts), search_nwb would be the fastest (or the only) tool that could be used. The search_nwb tool supports the most complete searches because it allows searching values not stored by nwbindexer and also allows searching DynamicTable layouts which are not searched by the NWB Query Engine.

### 3.7. Web Interface

A web interface is provided with the NWB Query Engines which enables client computer systems to run the tools through a web browser without installing them locally. This feature would be useful within a laboratory that uses multiple computer systems because the NWB Query Engines would only need to be installed once (on a system that has access to the NWB files) then the files can be searched from other computers in the lab using a web browser. It could also be useful for laboratories that use a remote (“cloud-based”) system to store data. In this case, the Query Engine tools would be installed on the server in the cloud hosting the data, and then could be searched by client systems in the lab using the web browser. (The shift of neurodata laboratories from locally maintained systems to cloud-based solutions is discussed in Rosenthal et al., 2010 and Vogelstein et al., 2016). The web interface could also be useful to enable search of files stored in neuroscience data repositories, for example, CRCNS.org^23^, G-NODE^24^, and EEGBase (Moucek et al., 2014).

**Figure 9:**
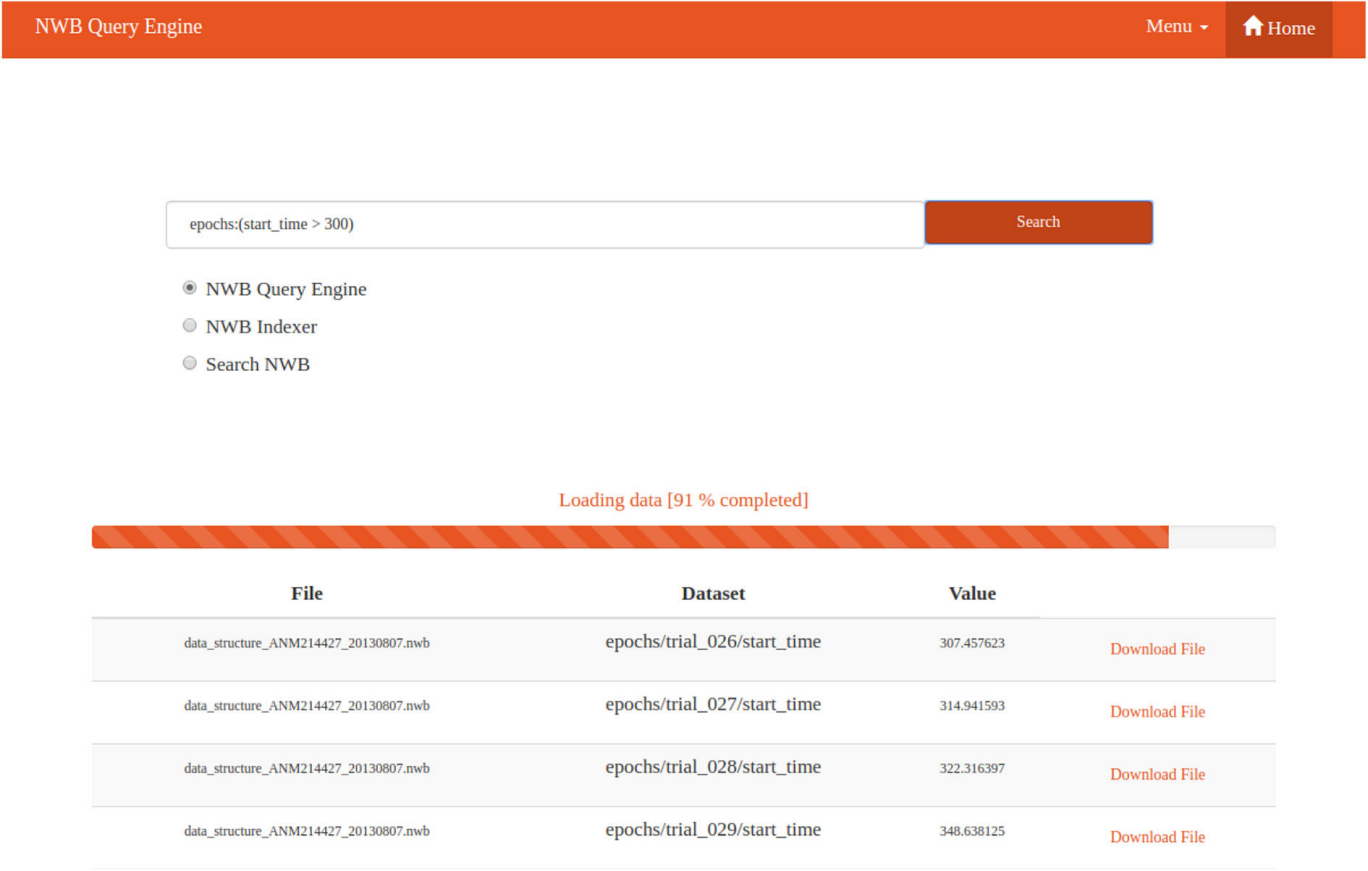
NWB Query Engine Web Interface Preview. The web interface provides a Google-like search box. The progress bar informs the user the percentage of searched files. A table with results is displayed piece by piece. A table row contains the name of the file with requested data, the name of the dataset in which data has been found, the value in the dataset, and a link for downloading the file.

A preview of the web interface is shown in Figure 9. It is a simple web page displaying basic information. The centerpiece of the web page is a search box for the user to input a query and select which tool should be used. When the user runs a query, the selected tool is called in the background. This call can be time consuming if a lot of data have to be searched. For this reason the web page is implemented dynamically, so that data are read and displayed incrementally as they are loaded. A progress bar informs the user how many data files have been searched. Once files matching the search are found the user can download them.

**Figure 10:**
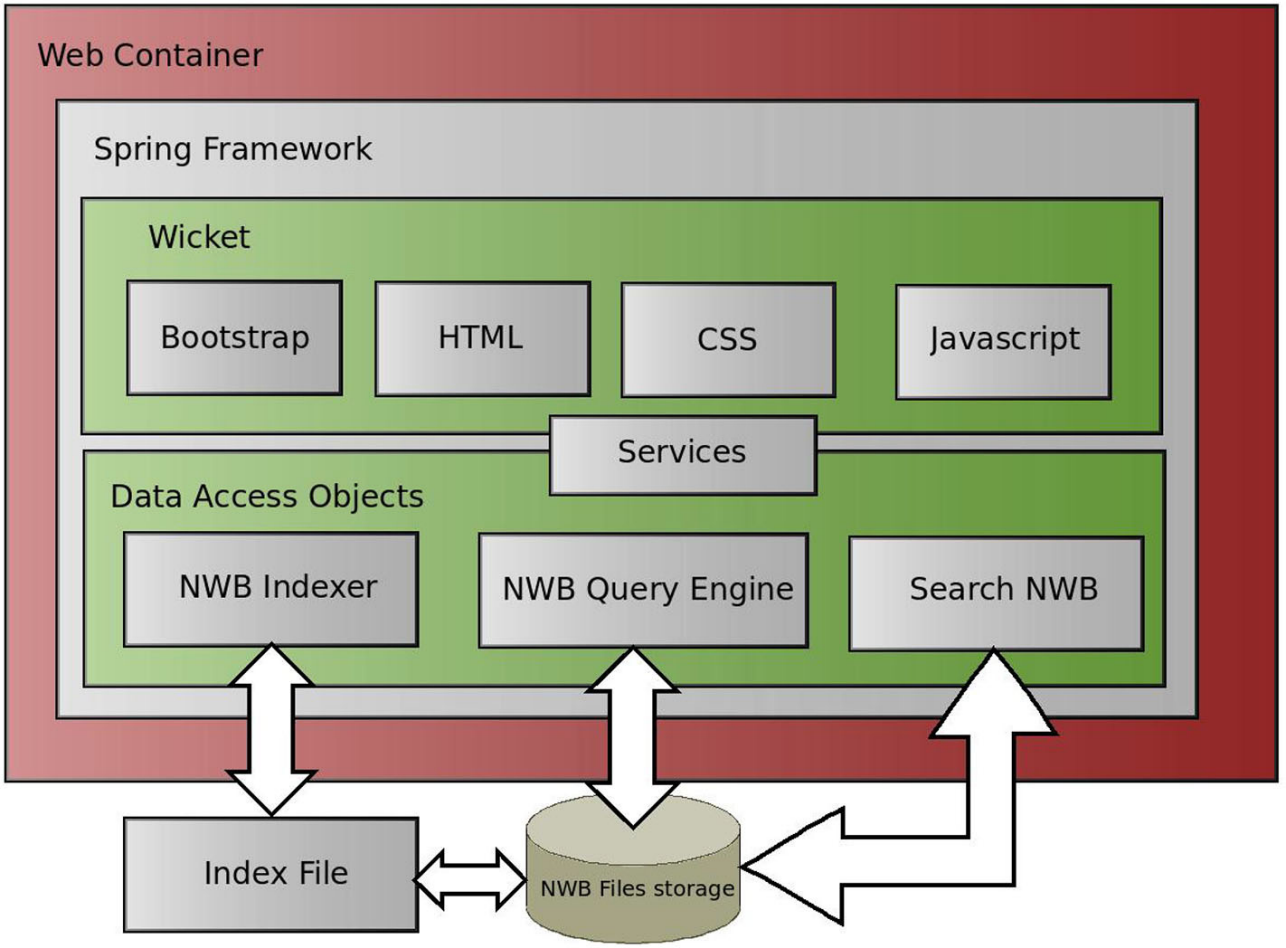
Implementation of Web Interface to the NWB Query Engines. The Web Interface is implemented in a common three layer architecture. We used the Spring Framwework. The data layer accesses data via the NWB Query Engine and returns results to the view layer via the service layer. The view layer is implemented in the Wicket framework.

The web Interface is implemented in the Spring framework (Johnson et al., 2004). It is an implementation of the Dependency Injection design pattern that allows developers to easily integrate individual modules to the core application. It also integrates other technologies, such as the Wicket framework, in user layer and an Ajax-based framework for the implementation of dynamic data loading. The design of the web pages is implemented in the Apache Bootstrap framework, a library of predefined CSS templates ready to be immediately deployed. A block schema of the web interface implementation is shown in Figure 10.

### 3.8. User Guide

All of the tools and the web interface are hosted in GitHub repositories^25^. The README file in each repository has installation instructions. The minimal requirement for running the NWB Query Engine is to have installed JRE 8^26^. When the query engine is called from a Python code a py4j python library^27^ is required in Python installation. The web interface runs in the Apache Tomcat^28^ web container. Since running and configuring a tomcat container could be an obstacle for some user, we also provided a docker^29^ file for easy build of a docker image. The search_nwb and nwbindexer tools require Python 3.7.

The output (search results) of the tools consist of the NWB file names, HDF5 path and values matching the search criteria. The format of the output varies slightly between the tools. The output of the search_nwb and nwbindexer tools is in JSON and is formatted to be human-readable^30^. The output of the NWB Query Engine is a human-readable table if it is run from a command line, but if the tool is used as a library called from a client application then that client application is responsible for formatting the search results. The output of all the three tools is unified in the web interface as a human readable table.

## 4. Discussion

The storage of neurophysiology data and metadata is complex, and there have been many systems developed to store such data. Systems that rely on a relational database to store the data (such as Datajoint, Yatsenko et al., 2015) allow searching the data because there is already a technology (SQL) developed for that purpose. However, for a variety of reasons, most systems for storing neurophysiology data use separate files which are organized so that the data recorded from a single session are stored independently of data recorded from other sessions. There is a need to be able to search collections of such files in order to find data of interest for a particular analysis. However, there does not yet exist any standard ways of implementing such searches.

We address this problem by presenting novel approaches and software to query neurophysiology data stored in separate files. The query tools we created are targeted for the recently developed Neurodata Without Borders format (Teeters et al., 2015; Rübel et al., 2017, 2019).

Because the NWB format is currently implemented using HDF5, our tools were designed to search within HDF5 files. Requirements for the search were based on the properties of the NWB format, which include storing data at locations for which the full path within the file may, or may not be, known in advance (described in section 2.2.1) and the storage of tabular data using different NWB-specific methods (describe in section 2.2.2) and for which data may be organized in multiple files using HDF5 external links (described in section 2.2.1).

Existing systems to search HDF5 files could not be used because most did not allow searching for data if the full path was not known in advance and none of them could be used to search data stored using the DynamicTable layout that is used in NWB 2 (described in section 2.2.2). In addition, none of the existing systems allow searching a collection of files. Instead, they only allow searching a single file.

The types of searches required, led us to define a syntactic structure of queries which has just two parts (a location, and an expression) separated by a colon. The syntax is much simpler than the SQL SELECT statements that are used for querying relational databases. An advantage of the query language presented here is that its use does not require any specific knowledge of database systems, SQL or even programming.

The query language we developed is similar to the DataJoint query language^31^ in the specification of the constraints on metadata values (called “operators” in DataJoint), but must be different in the specification of where in the file the metadata is located, because our tools are querying a hierarchical structure which does not have tables related by primary and foreign keys which are referenced in the DataJoint query language^32^.

**Figure 11:**
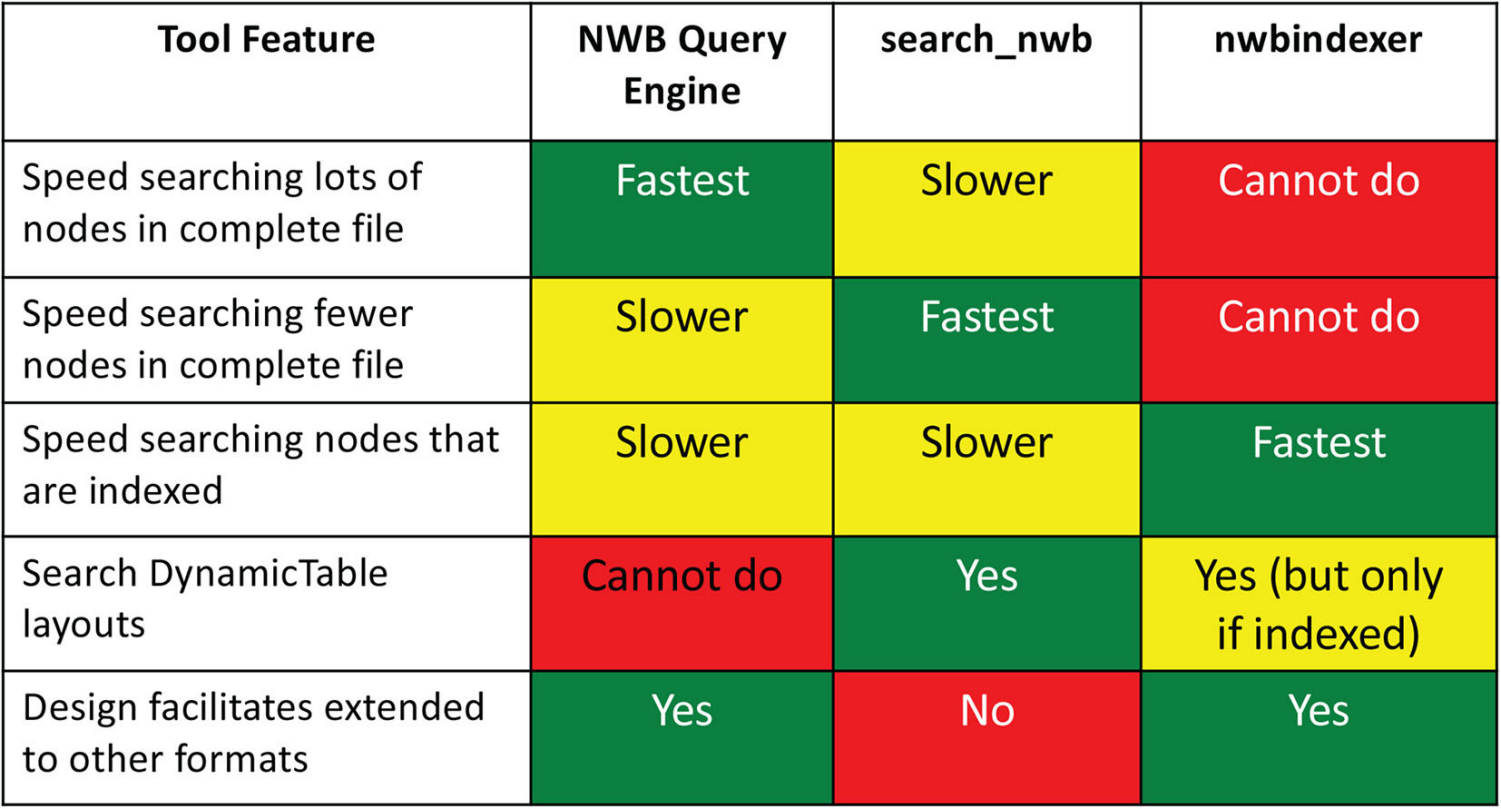
Comparison of tool features. Each tool is best for a particular purpose.

Three tools are presented. The first, the NWB Query Engine, is written in Java, but includes a Python and Web interface. It was originally designed to query files in NWB 1 but can also query files in NWB 2, but with some limitations on the search of tabular data. These limitations are overcome by the other two tools. The second tool, search_nwb, can query all data in both NWB 1 and NWB 2 files. The third tool, nwbindexer, operates in two steps: First a utility is run to build an SQLite database (index) containing the content of NWB files to be searched. Once this is done, queries are performed by a program that converts the queries into SQL which are then executed within SQLite. In many cases, these queries are faster than searches using the other tools. Since the SQLite database cannot contain all data in the NWB files the queries using nwbindexer search only a subset of the data. A comparison of the features of the three tools is shown in Figure 11.

Since these tools will normally be used to search metadata and not raw data, and the bulk of data in NWB files will usually be raw data (such as measurements of voltages or pixel intensities), typical searches using these tools can operate quickly even with very large file sizes because the raw data in the files will not be searched, only the much smaller metadata.

Even though the presented tools are specific to the NWB data format, two of them (the NWB Query Engine and nwbindexer) are implemented in a way that facilitates extending them to be used with other formats. In particular, the modular structure of the NWB Query Engine facilitates the extension to different types of data and formats for which separate files are used to store related data and for which the data within each file are organized in a hierarchical manner. Likewise, nwbindexer uses a single utility to build the SQLite database containing the data to be searched. This utility could be modified to read data from other hierarchically-organized formats and build an SQLite database for those. Once the SQLite database is created, the searches would be identical.

The tools we provide will work with NWB files regardless of how the files are created (whether they are created by Matlab, Python, or some other software). Installation of the tools only requires open source software (Java for one tool, Python for the others). The tools can be operated either from an interactive shell or from the web interface so the users are not required to be familiar with programming at all. The SQLite database used by the nwbindexer tool does not require any user effort to setup or administer since it is created automatically by running a single program (*build_index.py*).

Only the installation of the web interface will require technical knowledge. It is intended to be installed only once within a lab by a system administrator. We facilitate the installation by providing a docker file for building a transferable image. Installing the web interface is optional because it is not needed for running the tools.

Even though the tools are written in Java and Python they can be used from other languages. For example, programs written in Matlab can call Python functions, so Matlab code could also call any of the tools.

While the NWB format is evolving, both by changes to the core schema and also additions of extensions to the format, these tools should be sufficiently robust so to still work with future versions. This is because the tools do not depend on any particular NWB metadata, but they provide a framework (through the query language) to search any metadata. The user specifies the metadata when doing the search. The metadata to be searched is not hard-coded into the tools. In fact, an approach requiring hard-coding metadata into the tool would not work because it is impossible to know in advance what metadata will be included in extensions.

The tools operate on a single file system. However there are methods to connect individual data sources to a federated network, which would enable accessing distributed data as if they were on a single file system. Once data were federated in this distributed network all of the tools could operate on all files over the network. However, it is likely that only the nwbindexer tool would enable fast queries since the other two tools would require reading the NWB files over the network to do the search.

There are several assumptions that were made in creating these tools. One is that the user running the query must be familiar with how the data is stored within the NWB files, that is, within HDF5. Fortunately, for both NWB 1 and NWB 2, the layout of the data within HDF5 are described in the format documentation. Furthermore, there is a free utility called HDFView^33^ which allows a user to examine the contents of an HDF5 file in order to identify metadata that should be searched. So it should always be possible for users to know how data are stored in the files and thus create the queries.

A second assumption is that the NWB files will be using HDF5. This assumption is true with NWB 1 and is currently true with NWB 2. However, a goal of NWB 2 is to allow for other backend storage methods, not just HDF5. If the NWB files were stored in something other than HDF5, the tools presented here would need to be modified to read the data in the format they are stored.

## Data Availability Statement

The datasets referenced in this study are publicly available through the links provided.

## Author Contributions

PJ implemented the NWB Query Engine and the web interface. JT implemented the nwbindexer and search_nwb tools. FS supervised the project. PJ, JT, and FS wrote the manuscript. All authors contributed to the article and approved the submitted version.

## Conflict of Interest

The authors declare that the research was conducted in the absence of any commercial or financial relationships that could be construed as a potential conflict of interest.

## Footnotes

^1^https://www.brainproducts.com/filedownload.php?path=products/more/BrainVisionCoreDataFormat_1-0.pdf

^2^https://www.biosemi.com/

^3^https://alleninstitute.org/what-we-do/brain-science/

^4^Described in News section at http://nwb.org

^5^https://support.hdfgroup.org/HDF5/

^6^The latest version of the NWB format specification is at: https://nwb-schema.readthedocs.io/en/latest/.

^7^Even though the “string” data type may contain multiple characters, it is considered to be a scalar because in HDF5 strings can be stored using a pointer to the characters making up string, and the pointer is a scalar.

^8^http://www.hdfql.com/

^9^http://www.sqlite.org

^10^HDF5 Object references are similar to pointers in “C,” but they point to groups and datasets within an HDF5 file.

^11^In search_nwb and nwbindexer, object references are searched by converting each object reference to the path of the target (the path will be a string) and searching the string

^12^https://www.h5py.org

^13^https://docs.python.org/3/library/csv.html

^14^These thresholds, as well as others mentioned in this section, can be configured by constants in the top of the *build_index.py* program.

^15^Examples metadata of these different types from the queries in Table 1 are: start_time - scalar numeric; species - scalar string; tags - string array.

^16^https://www.nwb.org/example-datasets/

^17^http://crcns.org/data-sets/motor-cortex/alm-1

^18^File: Steinmetz2019_Forssmann_2017-11-05.nwb, Available at: https://figshare.com/articles/Datasets_from_Steinmetz_et_al_2019_in_NWB_format/11274968

^19^Available at: https://buzsakilab.nyumc.org/datasets/NWB/SenzaiNeuron2017/

^20^http://labshare.cshl.edu/shares/library/repository/37693/

^21^https://pynwb.readthedocs.io/en/latest/tutorials/index.html

^22^https://github.com/NeurodataWithoutBorders/pynwb/tree/fcb919a3b5c50a92c473e04a6a0b66f7e0204a27/docs/gallery

^23^http://crcns.org/

^24^http://www.g-node.org/

^25^NWB Query Engine: https://github.com/jezekp/NwbQueryEngine, nwbindexer & search_nwb: https://github.com/jeffteeters/nwbindexer, web interface: https://github.com/jezekp/nwbQueryEngineWebInterface

^26^http://www.oracle.com/technetwork/java/javase/downloads/jre8-downloads-2133155.html

^27^https://www.py4j.org/install.html

^28^https://tomcat.apache.org/download-80.cgi

^29^https://www.docker.com/

^30^The structure of the output is described in the online documentation at https://nwbindexer.readthedocs.io

^31^Described at https://docs.datajoint.io/python/queries/Queries.html

^32^While the nwbindexer tool does convert the hierarchical structure to a relational database, the resulting database cannot be queried by the DataJoint query language because the underlying relationships are still hierarchical. To query it, simple queries expressed in our query language must be translated to very complex queries in SQL (an example is given in Figure S6). It would be too complex (practically speaking, impossible) for a user to query the generated database directly without this automated translation step.

^33^https://www.hdfgroup.org/downloads/hdfview/
