# Supplementary material for: NWB Query Engines: Tools to Search Data Stored in Neurodata Without Borders Format
Source: Front Neuroinform. 2020 Sep 11;14:27. doi: 10.3389/fninf.2020.00027 (PMC7526650; doi:10.3389/fninf.2020.00027)
Supplement: Supplementary file 1 [file Presentation_1.pdf]

Supplementary Material: NWB Query Engines: tools to search data stored in Neurodata Without Borders format

1 DETAILS OF NWB QUERY ENGINE

This section provides details of the operation and implementation of the NWB Query Engine, including how a query is internally parsed, processed and evaluated, and how the tool can be called from Python code.

1.1 Operation modes

The NWB Query Engine can be operated in three ways:

1. Stand alone application - It is possible to run the tool as a command line application with two arguments. The first argument is a NWB file or a directory containing NWB files while the second one is a query. If a directory is used all NWB files within the directory are searched. The results are printed to a standard output.
2. Library function - The Query engine is integrated to a 3rd party tool. An input point is the *Input* interface provided by the NWB Query Engine. The client program only instantiate the *FileInput* class that enables reading NWB files from a file or a directory. Then only calls the method *executeQuery* from this interface. Results are returned in the *NWBResult* object. Easy integration with third-party tools is ensured by a Maven[1] artifact released and described in the project repository.
3. Python server - If the Query engine is run with a command line parameter *pyserver* it starts as a Python Gateway which can be called from a python code as described in Supplementary Material Section 1.3.

1.2 Parsing query

The parsed query is internally represented as a tree. An example is shown in Figure S1. The root node is an input query while other blue nodes are individual subexpressions. A pair of leaves connected by an operator represent a query filter. The left leaf represents the name of an attribute or a dataset and its right sibling represents a constant restriction. The parent of two sibling nodes stores an operator between those nodes. The NWB processor takes all leaves and starts evaluating them from down to top and from left to right (inorder). It starts processing the tree from the first left leaf that marks a group anywhere in the NWB file hierarchy. Then it continuously processes the descendants of the right sibling that represent subexpressions. Each subexpression is processed analogically. Moreover, if evaluation of a first expression returns an empty list the second expression is not evaluated if these are conjuncted (short-circuiting). This feature significantly increases the performance of the algorithm.

Let's say we have an extracellular electrophysiology recording of mouse doing a discrimination task with optogenetic and auditory stimulation. This experiment is composed from several trials where each of them takes some time and contains a different type of task.

[1] `https://maven.apache.org/`

In the example S1 the user wants to search all epochs in a time window 200s - 400s. The processor takes first left leaf: *epochs*. Then it traverses the left subtree of the right sibling and processes its leaves. First, it takes the left leaf and finds all datasets *start_time* in the *epochs* group. Second, it takes a constant *200* from right sibling and evaluates the condition given by the parent of these two leaves >. Last, if a result is found the right subtree is evaluated analogically. If not evaluation is terminated because the parent node of these two subtrees contains *and (&)* condition and short-circuiting is applied.

The user does not have to specify whether *epochs* is a group or a dataset or if *start_time* is a dataset or an attribute because the internal implementation searches for both by default. When a group *epochs* is found then the right side is supposed to be a dataset. If *epochs* were a dataset the right side would be interpreted as an attribute.

Figure S2 depicts an UML diagram of the complete implementation of the NWB Query Engine. The query parser block is implemented in the Query and QueryParser classes and the Parser interface. The NWBProcessor block is implemented in the Processor interface, and the NWProcessor and NWBResult classes. The FileApi block is implemented in the Input interface, and the FileInput and ArrayInput classes, and the Connector interface and the HDF5Connector class. The python server is implemented in the PyServer class. An application interface is represented by the Input interface. It has a query as an input parameter and returns a NWBResult object.

1.3 Python Gateway

The Python Gateway allows calling the Query engine from Python code without requiring knowledge about the internal implementation. It is implemented using a Python-Java bridge Py4j[2] which enables calling Java code from a Python program and executing a Java method defined as an entry point of this gateway. The entry point is the *Input* interface depicted in Figure S2.

When the NWB query engine is run as a Python Gateway then it can process queries coming from any python script. A code example is shown in Listing 1.

```
>>> from py4j.java_gateway import JavaGateway
>>> from py4j.java_gateway import GatewayParameters
>>> gateway = JavaGateway() # for localhost
>>> gateway = JavaGateway(gateway_parameters=GatewayParameters(address='remote host ip')) # or for
    remote host
>>> res = gateway.executeQuery("file or dir with nwb files", "query")
>>> for x in res:
...     print (x)
```

Listing 1: Example of calling the python server. In the first step a *JavaGateway* library is imported. Then, the user can call the *executeQuery* method with two parameters: (1) input file/dir and (2) required query. The final two lines iterate over the result and print the found datasets.

[2] https://www.py4j.org/

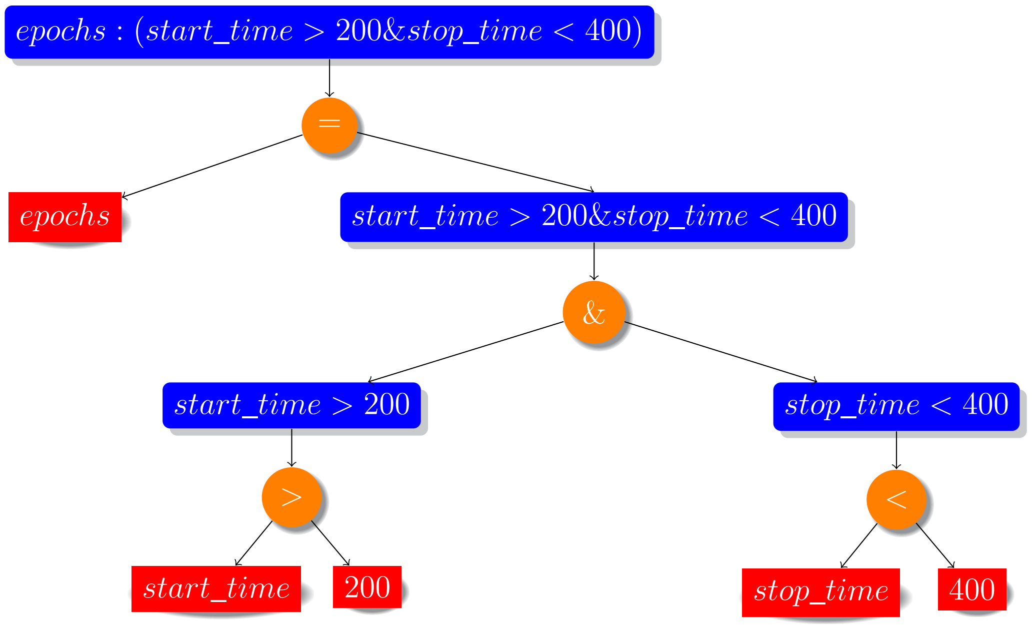

Figure S1: Query Grammar Tree Example

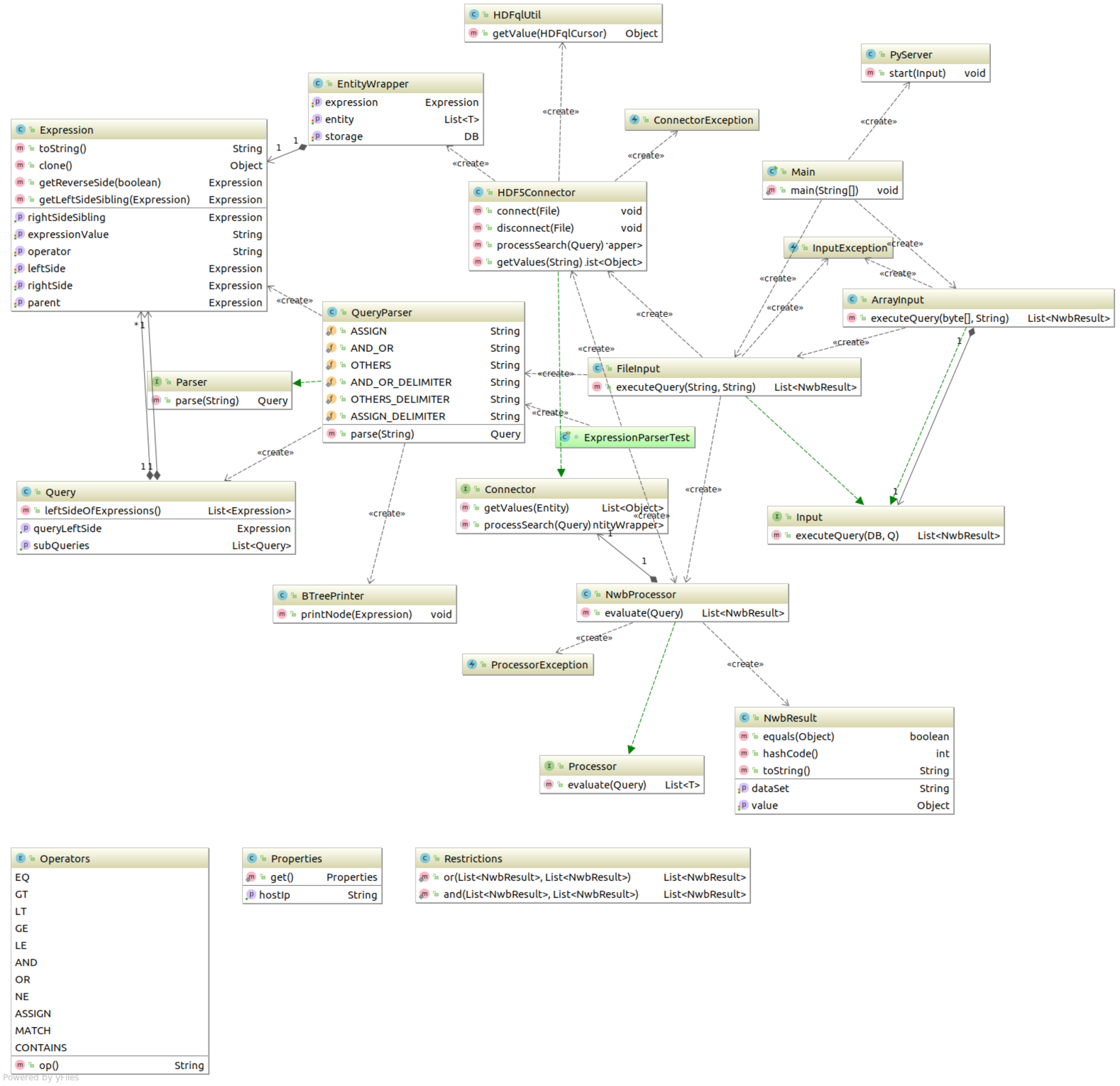

Figure S2: UML Diagram of the NWB Query Engine. A standard UML class diagram notation is used. Classes are marked by "C" letter in a blue circle. Interfaces are marked by "I" letter in a green circle. Solid lines with open arrowhead represent association. Green dashed lines with filled arrowhead represent inheritance. Black filled diamonds shapes at the end of solid lines represent a composition relationship. Gray dashed lines with open arrowhead represents a dependency.

2 DETAILS OF SEARCH_NWB IMPLEMENTATION

The following steps are used in the search_nwb utility (file *search_nwb.py*) to perform each query:

1. **Parse query.** The query is parsed using the Python “parsimonious” module[3]. File lib/parse.py contains the code to do the parsing. It contains a grammar for queries that is input to the parsimonious module and functions that create a Python dictionary containing information about the query. The dictionary is named "qi" (for "query information"). It is used in subsequent search steps.
2. **Iterate through NWB files.** The search_nwb tool has a required command line argument which is either the path to a directory containing NWB files, or a path to a single NWB file. If a directory is provided, the tool recursively scans the directory to find NWB files to search. If a path to a single NWB file is provided, that file is searched. In either case, the NWB file(s) to search are iterated over. Each file to be searched is opened for reading using the H5py library and the open file is used in the subsequent steps.
3. **Create and evaluate subquery call string.** The query specified to the search_nwb tool can be composed of multiple subqueries which are grouped using logical and (&), logical or (|) and also parentheses. The query (which is a string) is written following the grammar rules given in sections 3.2 and 3.4.1 of the main text. After parsing, information about the query is used to create a string, (called here, the "subquery call string") which contains a Python expression that can be executed using the Python "eval" function and which, when executed, will perform the query. The subquery call string is created by replacing each subquery in the query with a call to a function to execute the subquery, and by replacing the "&" and "|" operators by the Python logical "and" and "or" operators. Evaluating the subquery call string allows the Python interpreter to do short-circuit optimization in the execution of the subqueries; that is, to not execute a subquery if it’s determined that the subquery would not change the result of the overall query. The creation of the subquery call string is illustrated in Figure S3A-C. Each subquery calls function "runsubquery" which executes and saves the results of the subquery and returns Python logical *True* if the subquery succeeded, and *False* otherwises. The return values are used by the Python interpreter to do short-circuit optimizations.
4. **Find nodes that have all children referenced in subquery**. The first step of the "runsubquery" function is to search for nodes (groups or datasets) within the NWB file that have all the children that are referenced in the subquery. This is done by starting at the path specified for the parent in the subquery (left side of ":") and, if any wildcards are specified, doing a breadth-first search for nodes that match the parent path and which also contain all the children specified in the subquery. Each node that is found is passed to the next step.
5. **Search node children for matches to subquery constraints**. One of the requirements for the search_nwb utility is that the searches must work when the method of storing the data of children referenced in a subquery is mixed, that is, when the same subquery references a datasets that *is* a column in a DynamicTable and also a child that *is not* a column in a DynamicTable (the child could be an attribute). This requires two different methods of doing the search be performed within the same subquery: (i) The search for data that is not part of the DynamicTable must use a direct comparison of the values of the child with the specified constant given in the subquery without regard to the value of other variables in the expression. (ii) The search for data that is part of DynamicTable requires that the values of other variables in the expression be considered because for all children that are part of a

[3] https://pypi.org/project/parsimonious/

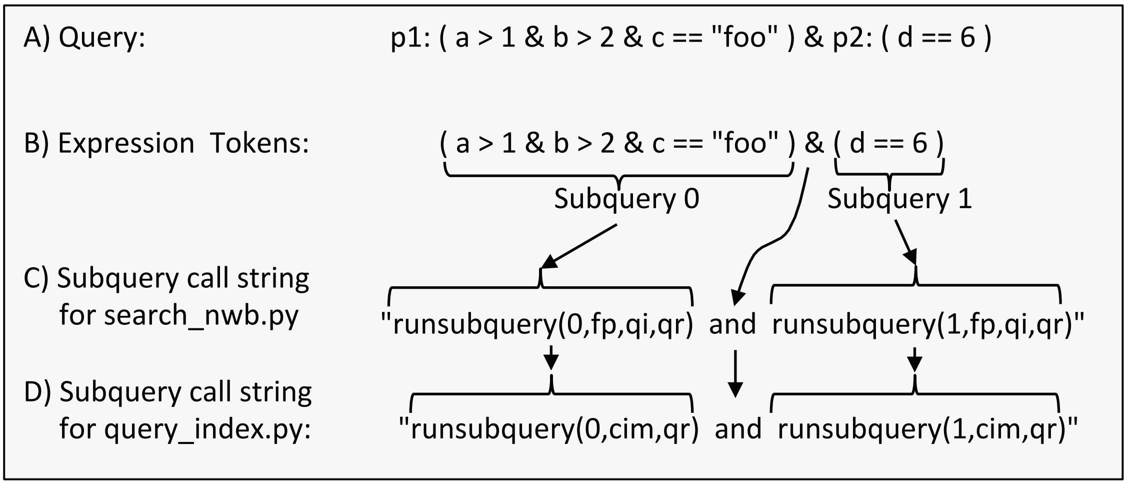

Figure S3: Creation of subquery call string for search_nwb tool. **(A)** Example query containing two subqueries. **(B)** The tokens making up the query expression, formed by removing all parts of each subquery to the left of the expression in the subquery. **(C)** To make the subquery call string, each expression in each subquery is replaced a call to function "runsubquery". For *search_nwb.py* the arguments are: the index of the subquery, 'fp' – file pointer (H5py File object) to the NWB file, 'qi' – query information (result of parse) and 'qr' – object for storing query results. **(D)** For *query_index.py*, the 'fp' and 'qi' arguments used above are replaced by 'cim' (a "Cloc_info_manager" object) which contains values of the children variables in both subqueries.

DynamicTable, the constraints specified in the subquery must be determined using values in the same row.

In order to fulfill this requirement, a two-phase process is used to check if a given node has values that match a subquery. These phases are implemented respectively by functions "get_individual_values" and "get_row_values" in file *search_nwb.py*. The first phase is to process all the children in the subquery that are not part of a DynamicTable. These are called "independent" children. This is done by considering each child individually and finding values that satisfy the binary expression referencing the child. After this is done, the original subquery is edited, replacing the binary expression making up the child with the string "True" if value(s) satisfying the expression were found, and the string "False" otherwise.

The first phase is illustrated in Figure S4, parts A-D. Parts A and B respectively show example values stored in children of a group and a subquery that searches for some of the values. (The children are "a"[4] which is not part of a DynamicTable, and datasets "b", "c_index" and "c" which are all part of a DynamicTable). Figure S4, part C shows that the search for the matching values for the independent child ("a") is done by loading all the values for "a" and creating a Python expression to evaluate using the Python 'filter' function which will return the value(s) that satisfy the expression. In part D, this Python expression is evaluated which returns an array containing the two matching values. These are saved in the query result object (qr). The subquery expression tokens are edited to replace the binary expression that references "a" with "True" since results were found. Otherwise, it would be replaced by "False". This edited subquery expression is used in the second phase, described below.

The second phase is to process the children that have values stored in a DynamicTable. This is done by further modifying the subquery expression made in the first phase to create a Python expression

[4] "a" would probably be an attribute since it's in a group that contains a DynamicTable but is not part of the DynamicTable. But it could also be a dataset.

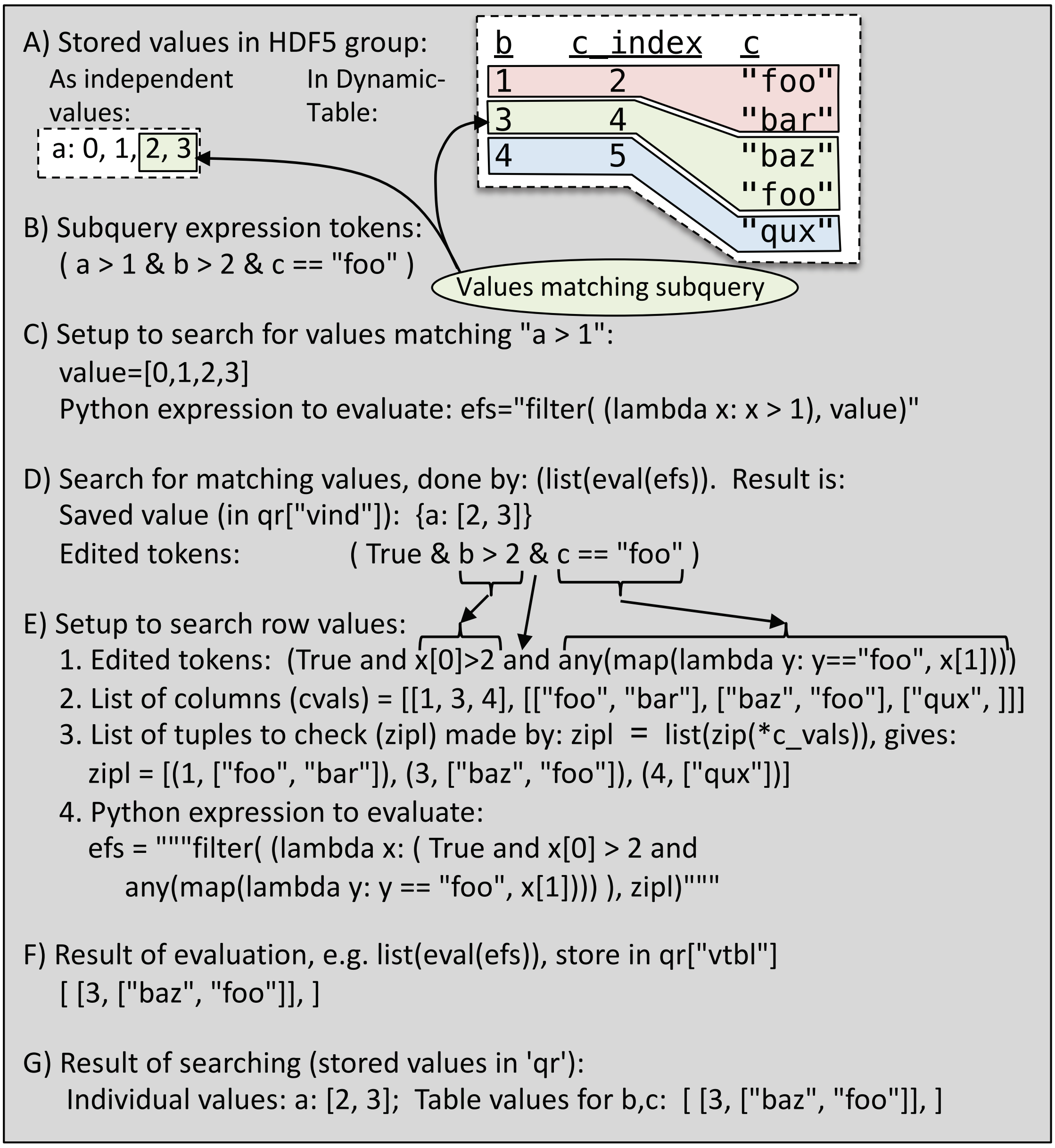

Figure S4: Steps used in search_nwb utility to search a node for values matching a subquery. **(A)** Example stored values for children in NWB group. **(B)** Example subquery expression. The values in green (above) match the expression constraints. **(C-D)** Steps used in first phase to search for matches to independent values (not part of a DynamicTable; in this case variable "a"). **(E-F)** Steps used in second phase to search for matches to values stored in DynamicTable layouts. **(G)** The found individual values and found row values are saved in the qr object. A more detailed description of the steps in this figure are given in Section 2 step 5.

that can be evaluated to find rows in the DynamicTable that result in the subquery expression being True. If found, the values in these rows are saved along with values found for individual children in the first phase.

This second phase is illustrated in Figure S4, parts E–G. In part E step 1, the expression tokens are edited again, replacing any variables that are stored in tables with either "x[n]" (if the values do not use an auxiliary index array) or a call of the form "any(map(lambda y: ... , x[n])" where "..." is

replaced by the binary expression containing the variable with "y" substituting for the variable name (if the values do use an auxiliary index array). In Figure S4 part E step 2 a list containing the columns in the DynamicTable is made and stored in variable "cvals"[5]. Each element of a column that has an associated index array contains a list of the values in the corresponding row (since, if the column has an associated index array, there may be multiple values stored in each row of that column). In Figure S4 part E step 3, a list of tuples to check (stored in variable "zipl") is made where each element is the list of the values of all variables in the same row. Those values that have an auxiliary index array will also be lists. List "zipl" is created using the Python assignment zipl = list(zip(*cvals)) which converts a list of lists stored in variable "cvals" into a list of tuples containing aligned values from the lists, (e.g. if cvals = [["a", "b", "c"], [1, 2, [3, 4]]]); zipl = list(zip(*(cvals))), gives: [('a', 1), ('b', 2), ('c', [3, 4])]). Figure S4 part E step 4) The edited tokens is put into a string, (named 'efs' in the figure) which contains a call to the "filter" function as in part C. In Figure S4 part F the 'efs' string is evaluated returning the matching row values. G) The found individual values and found row values are saved in the qr object.

3 DETAILS OF NWBINDEXER IMPLEMENTATION

This section provides details of the SQLite3 database made by the nwbindexer *build_index.py* utility and how the database is searched by the *query_index.py* utility.

3.1 Specification of node_type in SQLite node table

As illustrated in the main text, Figure 6, the node table has field named "node_type" which stores a single character. The value of the field indicates the type of node. Possible values and the meaning are: **a** - attribute; **d** - dataset; **g** - group that does not contain a "colnames" attribute (thus does not contain a DynamicTable); **G** - group containing a 'colnames' attribute (e.g. the group contains a DynamicTable). (The DynamicTable layout is described in Section 2.2.2 of the main text.)

3.2 Storage of Values in SQLite database

As mentioned in the main text, Section 3.5.3, value table nval field stores only a single numeric value (integer or float) and the sval field store all other types of data including strings, string arrays, and numeric arrays that are in a DynamicTable.

In the value table, the type of the value ("type" field) indicates what type of data is stored. The possible types are grouped according to whether or not the values are a column in a DynamicTable and whether or not an index dataset is used. The different types within each of these groups are:

- *Not column in a DynamicTable*: scalar integer (**i**), scalar float (**f**), scalar string (**s**), string array (**S**).
- *Column in DynamicTable, not using index array*. Integer array (**I**), float array (**F**), string array (**M**), compound (**c**).
- *Column in DynamicTable, using index array*. Integer array (**J**), float array (**G**), string array (**B**) and compound (**c**).

For values that are in a DynamicTable, but not type "c", the values are stored using the following format in the "sval" string:

<column_values><index_values>

[5] "cvals" stands for "column values". It is not related to the variable "c" in Figure S4.

Where *<column_values>* is the CSV list of values in the column and *<index_values>* (if present) contains the index values in csv form prefixed by "i".

Type "c" is used for both HDF5 compound types and also for HDF5 2-d datasets. For type c, the column types and the presence or absence of the index array are indicated by the following format of the "sval" string:

<column_types><index_flag><column_names>,<column_values><index_values>

Where: *<column_types>* is a string made up of one character per column with each character the type of the corresponding column as specified above. In other words, each character of *<column_type>* will be either I, F, M (if an index dataset is not used) or J, G or B (if an index dataset is used). *<index_flag>* is either "n" if there is no index array, or "i" if an index array is present. It is present not only to indicate whether or not there is an index array, but also to mark the end of the *<column_types>*, since if no character was present, the first character of *<column_names>* could be mistaken for a part of *<column_types>*. *<column_names>* is a CSV list of the column names if the table is a HDF5 compound type, or a CSV list of the zero-based column indices (e.g. 0,1,2, ...) if the value is a HDF5 2-d dataset. *<column_values>* is the csv list of values in all columns concatenated together. *<index_values>* (if present) contains the index values in csv form, with a prefix "i". The prefix "i" serves to separate the index values from end of the *<column_values>* which could be integers.

As described above, values of index datasets in a DynamicTable are stored by appending the values in CSV form to the values of the data set that they provide the index for. This allows the *query_index.py* utility to retrieve the values of both the target dataset and the index dataset (which are used to interpret the values) as one string, which can then be unpacked and used.

Examples of values stored are shown in Figure S5.

3.3 Implementation of *build_index.py* utility

To reduce the time required to build the database, Python dictionaries are used as caches within the *build_index.py* program to speedup the lookup of contents previously saved in the SQLite database.

3.4 Implementation of *query_index.py* utility

The following steps are performed by the *query_index.py* utility:

1. **Parse query**. This is the same as step 1 used in the implementation of the search_nwb utility, described in Section 2 of this Supplementary Material.
2. **Create and execute SQL queries**. For each subquery in the specified query, create and execute two SQL queries (SELECT statements): a "normal" query which searches all files for all nodes (groups and datasets) that satisfy the subquery expression, and for which the parent node is not a group that contain a DynamicTable; and a "table" query, which searches all files for groups that contain a DynamicTable and which also contain all children referenced in the subquery. The creation and execution of these SQL queries is done when creating an instance of the "Cloc_info_manager" class. An instance of this class is created in function "perform_query" and stored in variable named "cim". The SQL queries are created by function "make_sql" in file "lib/make_sql.py". Examples are shown in Figure S6. The result of executing the SQL queries are stored in the "sqr" instance variable which is a Python dictionary that stores the data returned by the queries indexed by the file_id, and within that, indexed by the subquery and within that, indexed by nodes (paths) found within each subquery. The end result is that all of

the data needed to compute the query results are saved in the "sqr" variable and this data is organized by the file_id, subquery number, and path within the file of the parent node and it contains values of children that may satisfy the constraints on each subquery.

3. **Iterate through files**. This is done by iterating through the files that have results found in the SQL select statements executed in step 2 (stored in variable cim.sqr). For each file:
4. **Create and evaluate a "subquery call string."** The subquery call string created is illustrated in Figure S3 part D. It is similar to the subquery call string used in the search_nwb utility (shown in Figure S3 part C), but has different calling parameters. Specifically, the first parameter (subquery index) and last parameter (qr – query result) remains the same, but the middle two parameters fp – file pointer (a H5py File object) and qi – query information are replaced by "cim" (the "Cloc_info_manager" object created in step 2. Evaluating the subquery call string (using the Python "eval" function) results in, function "runsubquery" being called to perform each subquery. Using the eval function causes the Python interpreter to short-circuit (not make unnecessary calls) when possible.
5. **Iterate through parent nodes**. This is done in function runsubquery by iterating through the nodes found by the SQL queries in step 2. For each parent node the next step (below) is done.
6. **Find values of children that satisfy subquery constraints**. The children values are obtained by calling function cim.get_children_info(). The values satisfying the constraints given in the subquery are found using the methods used in the search_nwb utility described in section 2 step 5. Note, that in Figure S4, the value of variable "a" would not be as shown in the figure because the *build_index.py* program would not store the values for numeric arrays that contain more than one element and are not a column in a DynamicTable. But if variable "a" contained a scalar value or was an array with only one element, that would be stored in the SQLite3 database.

A flow chart comparing the operation of *search_nwb.py* and *query_index.py* is shown in Figure S7.

3.5 Alternate designs considered

When designing the database schema for nwbindexer some alternative designs were considered. One was to eliminate the name table and have the path for all HDF5 entries that are in the node table (groups, datasets and attributes) be stored in the path table. This took up more space and made the SQL queries less efficient. Another was to eliminate the path table and use only the name table to store path information and use SQLite recursive queries (also called Common Table Expressions, or CTE) to build paths that could be searched as part of the SQL select statements. This made the queries too slow.

Other possible designs were considered for storing the values. Instead of packing array values into CSV strings, an alternative approach that had a table of strings and another table for numeric values was considered, with each array element stored as a separate row entry in the proper table and with an id column used to keep track of the array indices. Prototypes showed this could work and that queries could be done in SQL to find matching values to queries even with the index array used with a DynamicTable, however, with a large (realistic) amount of data, queries became quite slow; plus it would require more space to store the values. Another alternative design concerned the generated SQL code. Instead of having two SQL Select statements ("normal" and "table", described in Section 3.4 part 2) a method to have a single SQL Select statement was tested using the SQLite "case" function to incorporate the functionality of both into a single Select statement. This was more complex than having two SQL Select statements and took much longer to execute.

A	Data stored not part of DynamicTable	Contents of value table:		
**Example id**	**Value to store**	**type**	**nval**	**sval**
A1	47 (integer)	n	47	
A2	5.7 (float)	f	5.7	
A3	"mouse" (string)	s		mouse
A4	"foo","bar","baz" (string array)	S		foo,bar,baz

B	Data stored in DynamicTable, without index values	Contents of value table:		
**Example id**	**Value to store**	**type**	**nval**	**sval**
B1	"foo","bar","baz" (string array)	M		foo,bar,baz
B2	3,7,14 (integer array)	I		3,7,14
B3	3.5, 2.0, 6.7 (float array)	F		3.5,2,6.7
B4	A b c (Compound or 2-d dataset)<br>foo 1 0.7<br>bar 3 1.2	c		See below.
	sval: `MIFnA,b,c,foo,bar,1,3,.7,1.2`			

C	Data stored in DynamicTable, with index values	Contents of value table:		
**Example id**	**Value to store**	**type**	**nval**	**sval**
C1	"foo","bar","baz" (string array); 1,3 (index array)	B		foo,bar,bazi1,<br>3
C2	3,7,14 (integer array); 1,3 (index array)	J		3,7,14i1,3
C3	3.5, 2.0, 6.7 (float array); 1,3 (index array)	G		3.5,2,6.7i1,3
C4	a b c (Compound or 2-d dataset;<br>1 0.7 foo 1,2 index array).<br>3 1.2 bar	c		See below.
	sval: `JGBia,b,c,1,3,.7,1.2,foo,bari1,2`			

Figure S5: Example values stored in SQLite database. For all panels **(A-C)** the type of data is indicated by the "type" field and values in the "sval" field are stored in CSV format. **(A)** If the data is not part of a DynamicTable, only scalar numeric values are stored (in the nval field) and strings and string arrays (which are stored in the sval field). **(B & C)** Data that are part of a DynamicTable are all stored in the sval field. Different types codes are used to indicate the type of data and whether or not an index array is present. Compound or 2-d datasets are indicated by type code "c" and with the individual column types and column names proceeding the values in sval. **(B)** If the DynamicTable does not have an index array, type codes "M", "I", "F" and "c" are used. If type "c", the <*index_flag*> is "n". **(C)**: If the DynamicTable does have an index array, then type codes "B", "J", "G" and "c" are used. For type "c" the <*index_flag*> is "i" and the index array values (with a prefix 'i') are appended to the end of sval. See Section 3.2 for more details.

A. Normal Query

```
SELECT
    f.id as file_id,
    f.location as file,
    bap.path as parent_a,
    ban.node_type as node_type,
    'species',
    ba0n.node_type as node_type,
    ba0v.type as type,
    ba0v.sval as species_value
FROM
    file as f,
    node as ban,
    path as bap,
    value as ba0v,
    node as ba0n,
    name as ba0na
WHERE
    bap.path LIKE 'general/subject'
        AND
    bap.id = ban.path_id AND
    f.id = ban.file_id AND
    ba0n.parent_id = ban.id AND
    ba0n.name_id = ba0na.id AND
    ba0na.name = 'species' AND
    ba0v.id = ba0n.value_id AND
    (ba0v.sval LIKE "%mouse%") AND
        ban.node_type != 'G'
```

B. Table Query

```
SELECT
    f.id as file_id,
    f.location as file,
    bap.path as parent_a,
    ban.node_type as node_type,
    'species',
    ba0n.node_type as node_type,
    ba0v.type as type,
    case when ba0v.type in ('i', 'f') then ba0v.
    nval else ba0v.sval end as species_value
FROM
    file as f,
    node as ban,
    path as bap,
    node as ban_colnames,
    name as bana_colnames,
    value as bav_colnames,
    value as ba0v,
    node as ba0n,
    name as ba0na
WHERE
    bap.path LIKE 'general/subject' AND
    bap.id = ban.path_id AND
    f.id = ban.file_id AND
    ban_colnames.value_id = bav_colnames.id AND
    ba0n.parent_id = ban.id AND
    ba0n.name_id = ba0na.id AND
    ba0na.name = 'species' AND
    ba0v.id = ba0n.value_id AND
        ban_colnames.parent_id = ban.id AND
        ban.node_type = 'G' AND
        ban_colnames.node_type = 'a' AND
        ban_colnames.name_id = bana_colnames.id
    AND
        bana_colnames.name = 'colnames'
```

Figure S6: Sample SQL queries generated by nwbindexer. The SQL queries are generated for query: /general/subject: species == "mouse". **(A)** The normal query searches for nodes (groups, datasets and attributes) that satisfy the subquery expression, and for which the parent node is not a group that contain a DynamicTable. Variable "ban" is an alias for the parent node, and the constraint at the end (ban.node_type != 'G') prevents selecting results where the parent node is a group containing a DynamicTable (possible values for node_type are given in section 3.1). **(B)** The "table" query, searches for groups that contain a DynamicTable and which also contain all children referenced in the subquery. **(A)** & **(B)** In both queries, the table aliases (except for the file table) starts with: "b<subquery_char><child_index>" where <subquery_char> is: "a"-first subquery, "b"-2nd subquery, ...; and <child_index> is not present if the node is a parent, otherwise, it's: 0-first child, 1-second child, and so on. The alias suffix indicates Which table is being referenced: "n" - node table, "p" - path, "v" - value, "na" - name. The string "_colnames" is appended to the alias in the Table Query for the node, name and value tables used to retrieve the value of the "colnames" attribute in a group that contain a DynamicTable.

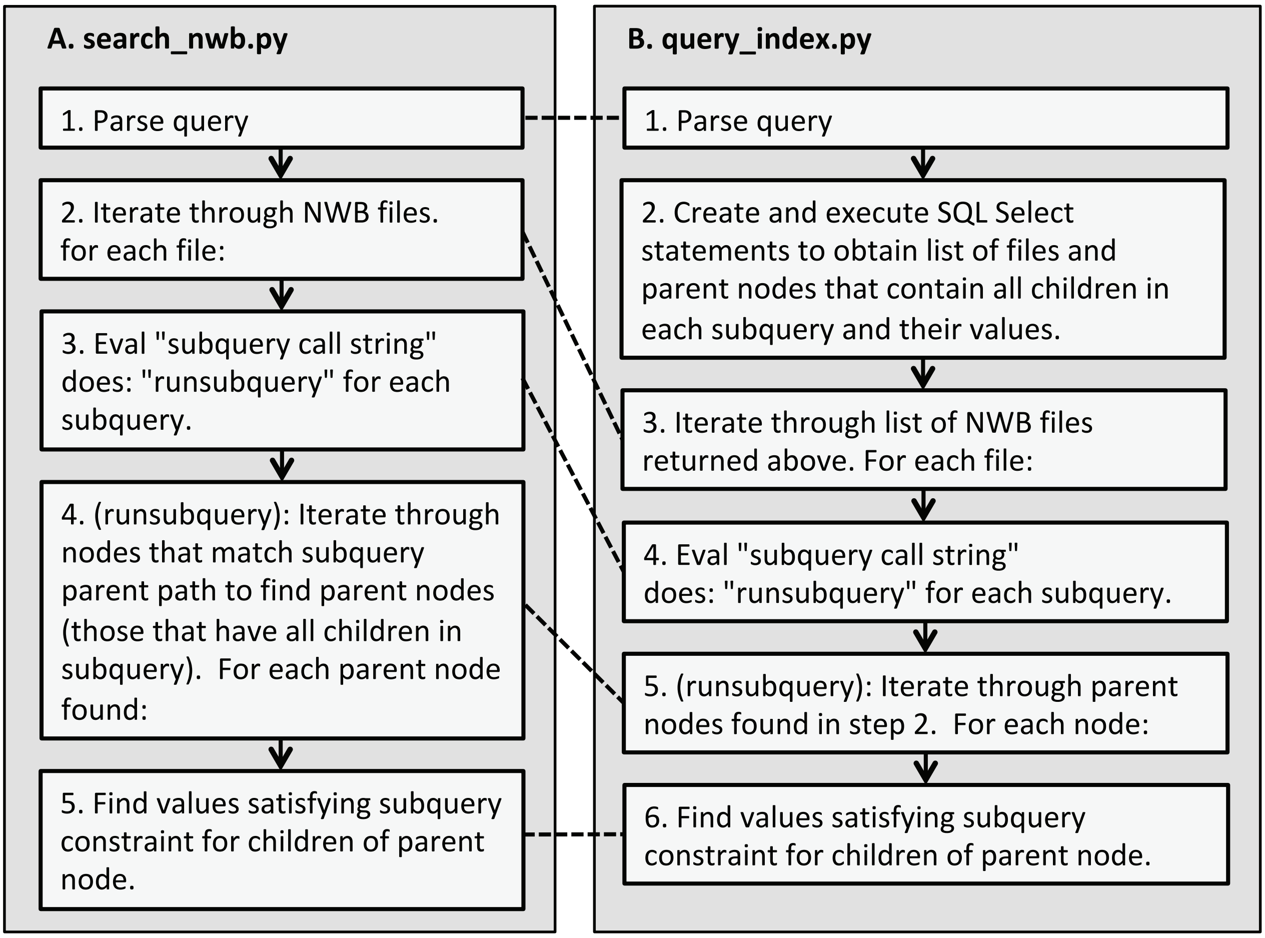

Figure S7: Comparison of steps used to execute a query by the *search_nwb.py* and *query_index.py* utilities. The dashed lines between boxes indicate steps that perform similar functions.
